# Inhomogeneity Based Characterization of Distribution Patterns on the Plasma Membrane

**DOI:** 10.1371/journal.pcbi.1005095

**Published:** 2016-09-07

**Authors:** Laura Paparelli, Nikky Corthout, Benjamin Pavie, Devin L. Wakefield, Ragna Sannerud, Tijana Jovanovic-Talisman, Wim Annaert, Sebastian Munck

**Affiliations:** 1 VIB Bio Imaging Core, Herestraat, Leuven, Belgium; 2 Laboratory of Membrane Trafficking, Department of Human Genetics, KU Leuven, Herestraat, Leuven, Belgium; 3 VIB Center for the Biology of Disease, KU Leuven, Herestraat, Leuven, Belgium; 4 VIB, LiMoNe, Herestraat, Leuven, Belgium; 5 Department of Molecular Medicine, Beckman Research Institute of the City of Hope Comprehensive Cancer Center, Duarte, California, United States of America; Icahn School of Medicine at Mount Sinai, UNITED STATES

## Abstract

Cell surface protein and lipid molecules are organized in various patterns: randomly, along gradients, or clustered when segregated into discrete micro- and nano-domains. Their distribution is tightly coupled to events such as polarization, endocytosis, and intracellular signaling, but challenging to quantify using traditional techniques. Here we present a novel approach to quantify the distribution of plasma membrane proteins and lipids. This approach describes spatial patterns in degrees of inhomogeneity and incorporates an intensity-based correction to analyze images with a wide range of resolutions; we have termed it **Qu**antitative **A**nalysis of the **S**patial distributions in **I**mages using **Mo**saic segmentation and **D**ual parameter **O**ptimization in **H**istograms (QuASIMoDOH). We tested its applicability using simulated microscopy images and images acquired by widefield microscopy, total internal reflection microscopy, structured illumination microscopy, and photoactivated localization microscopy. We validated QuASIMoDOH, successfully quantifying the distribution of protein and lipid molecules detected with several labeling techniques, in different cell model systems. We also used this method to characterize the reorganization of cell surface lipids in response to disrupted endosomal trafficking and to detect dynamic changes in the global and local organization of epidermal growth factor receptors across the cell surface. Our findings demonstrate that QuASIMoDOH can be used to assess protein and lipid patterns, quantifying distribution changes and spatial reorganization at the cell surface. An ImageJ/Fiji plugin of this analysis tool is provided.

## Author Summary

Plasma membrane organization is fundamental to cellular signaling, transport of molecules, and cell adhesion. To achieve this, plasma membrane proteins and lipids are spatially organized: they form clusters, aggregate in signaling platforms, distribute into gradients on polarized cells, or randomly distribute across the membrane. It is also clear that these organizations can be affected in various contexts. For example, in aging or neurodegenerative diseases, the composition of the plasma membrane is altered and, consequently, the protein and lipid distributions in the membrane fluctuate. In addition, cancer progression is characterized by changes in cellular polarity, lipid content, and the redistribution of cell surface receptors and adhesion molecules. Here we have developed a method to quantify such alterations that, unlike current tools, is compatible with diverse types of cellular organization, including polarity. Our tool can be employed to screen for changes in a straightforward manner and to elucidate distributions of cell surface components in different disciplines, ranging from neurobiology to cancer research.

## Introduction

The function of cell surface proteins and lipids is tightly coupled to their spatial organization [1–3]. Membrane constituents cluster in nano- and micro-domains originating from lipid affinity (e.g., lipid rafts) [4], protein-protein interactions (e.g., tetraspanin domains) [5], and constraints imposed by the cytoskeleton [6]. Plasma membrane organization is also inherently asymmetric in polarized cells, such as migrating cells [7,8], epithelial cells [9], and neurons [10,11]. The ability to detect this plasma membrane organization is crucial for unraveling the dependency of signaling events, and understanding membrane regulation in a disease-related context [12].

One approach to assess the distribution of plasma membrane molecules is to consider these as bi-dimensional point processes that can be analyzed by spatial statistics. Point processes can be classified as: (I) Homogeneous or random, characterized by a constant density of points; (II) Inhomogeneous, characterized by a non-constant density of points; (III) Regular, with points equally dispersed; and (IV) Clustered, where points are grouped [13].

For investigating the spatial organization and especially for studying clusters, Ripley’s K-function [14] and pair correlation (PC) function approaches have been established. These measure the number from neighbors within a certain distance of a protein to determine the amount of clustering [15,16]. Several modifications and extensions of Ripley’s K-function have been made, including a model-based Bayesian approach [17], the extension to co-clustering [18], and an adaptation to account for limited localization precision in single molecule localization microscopy [19]. The PC function has been applied to images acquired by Photoactivatable Localization Microscopy (PALM) to quantify the heterogeneity of protein distributions on the plasma membrane [20]. In addition, the Density-Based Spatial Clustering of Applications with Noise (DBSCAN) algorithm, a density based tool, was used to identify clusters of varying shape against a background in super-resolution microscopy [21]. Recently, the Ordering Points To Identify the Clustering Structure (OPTICS) algorithm was made available to the single molecule community to measure local density changes (overcoming some of the limitations of DBSCAN) [22,23]. Moreover, the Getis-Ord G statistic [24] has been used to quantify the degree of local protein clustering in super-resolution images [25] and compared to other methods including DBSCAN and PC analyses.

A key limitation of the current toolset is the lack of assessment on both clustering and polarity, equally important from a biological point of view. While the ability to detect specific clusters is of great importance, Sengupta et al. [20] among others, have shown that molecules like glycosylphosphatidylinositol (GPI)-anchored proteins are organized into different populations of clusters as well as residing as single molecules. All these organizations should be accounted for when analyzing the overall distribution. Furthermore, current tools require knowledge of the precise localization of the proteins and are thus limited to super-resolution or electron microscopy images. Therefore, we aimed to create an analysis tool that investigates biomolecule distributions without *upfront* information about their organization, and that can be applied to a variety of microscopy methods, including super-resolution and widefield microscopy. The main principle of our approach is to investigate spatial patterns of proteins and lipids and to quantify any deviation from random towards clustered or polarized organization as a measure of increased inhomogeneity.

We exploit a geometrical approach, called tessellation, to divide the image into tiles, and use the distribution of tile areas to characterize different patterns. The image analysis algorithm uses both the information of the neighbor relations of segmented objects and the intensity of the tiles in which they are confined. By using fluorescence intensity information for tile area correction, we have made the tool applicable to images acquired by a range of microscopy techniques. We have termed this analysis tool **Qu**antitative **A**nalysis of the **S**patial-distributions in **I**mages using **Mo**saic segmentation and **D**ual parameter **O**ptimization in **H**istograms (QuASIMoDOH). An ImageJ/Fiji [26] plugin for QuASIMoDOH analysis is available along with instructional documentation (S1 File).

## Results

### QuASIMoDOH analysis principle

For QuASIMoDOH analysis we consider single fluorescent emitters distributed on the cell surface as a point process *P* in a bi-dimensional space. Considering a number *N* of individual points *p* of the process placed on the support *S*, the distribution pattern *P* is defined as:

$$P=\sum_{i=1}^{N}p_{i}\text{ with }p_{i}\;\in \;S,\forall i\in \left\{1,2,3\ldots N\right\},$$ (1)

where *P* describes a homogenous, clustered, or inhomogeneous pattern. Considering the brightness *f* of the fluorescent emitters (represented by *p*) and assuming the emitters being equally bright, the distribution pattern can be defined as:

$$P=\sum_{i=1}^{N}p_{i}f\text{ with }p_{i}\;\in \;S,\;\forall \;i\;\in \left\{1,2,3\ldots N\right\}.$$ (2)

When the point process *P* (S1A Fig) is imaged by an optical system, each point of position *r*, *p*(*r*), will be diffracted by the Point Spread Function (PSF). The resulting microscope image *g*(*r*) is typically approximated by the convolution (⊗) of the point *p*(*r*) with the PSF [27]:

g(r)=p(r) ⊗ PSF(r). (3)

The result is a blurred image (S1B Fig). Due to the band limitation of the PSF, points of the pattern *P* placed at a distance shorter than the band limit will not be resolved as single points. In the blurred image, the intensity of the pixels depicting the diffraction pattern is directly related to the number of points contributing to this diffracted pattern.

**Fig 1:**
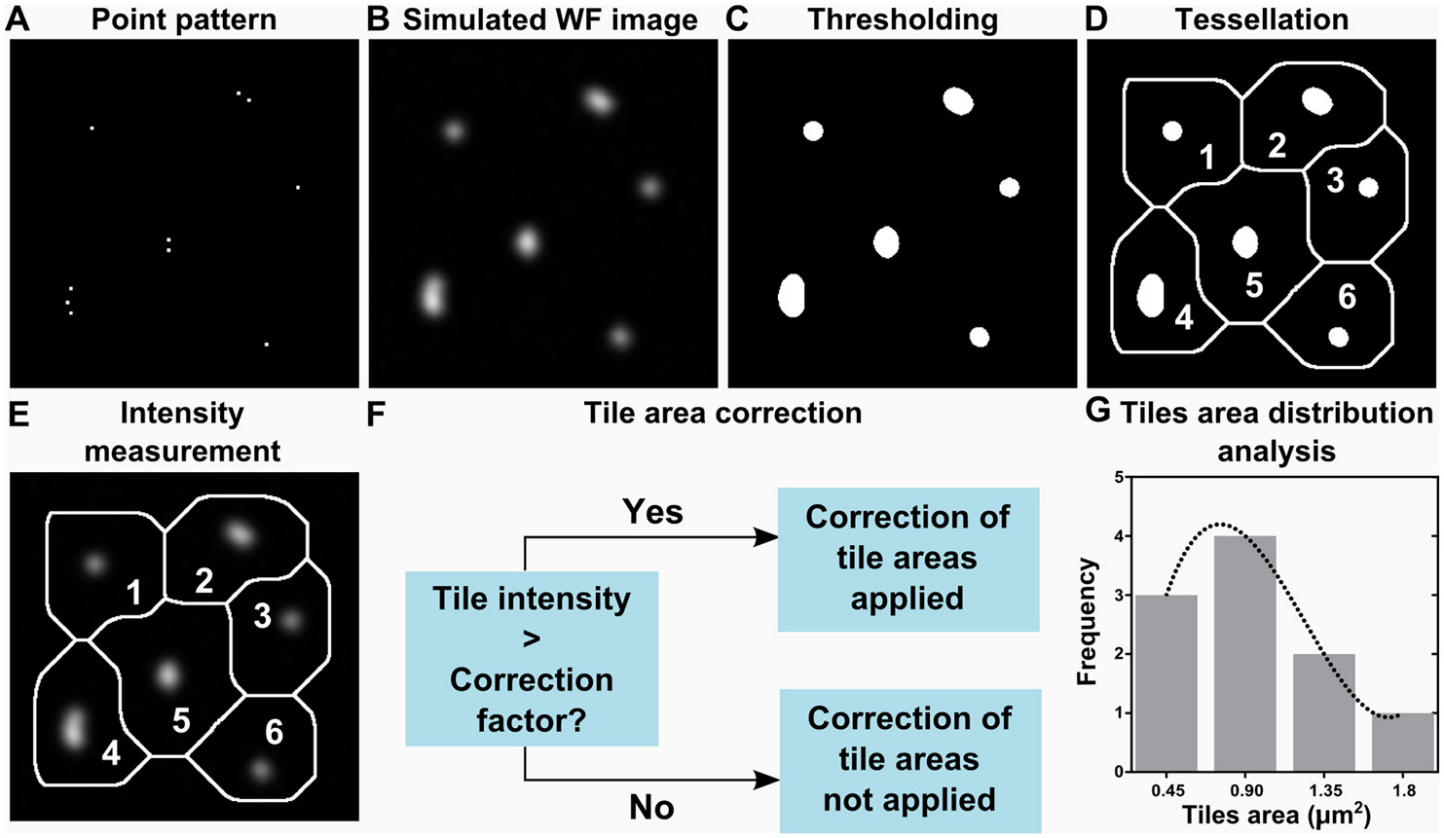
QuASIMoDOH analysis steps. (A) Computer generated image of an arbitrary point pattern. (B) The widefield microscope acquisition process is simulated. Blur by a point spread function and noise are introduced in the image (A) as shown in S1 Fig. (C) Thresholded image (threshold ‘Default’ was selected from the list of ImageJ/Fiji automatic thresholds). (D) Tessellation of the thresholded image; a number has been assigned to each tile. (E) The tile set generated from the binary image in (C) is applied to the grayscale image in (B) for measuring the intensity of the individual tiles. (F) Schematic of the tile area correction. (G) Histogram of tile areas and a curve fitting of the tile area distribution.

Aiding the development of QuASIMoDOH, we generated *in silico* images of points (shown as single pixels) dispersed on a surface (Fig 1A, S1 Text). Blur and noise were then added into the image (Fig 1B) to mimic the acquisition process of a fluorescence microscope (see S1 Fig and S1 Text for detailed description). The individual steps of QuASIMoDOH analysis are depicted in Fig 1 and described in detail below:

#### 1. Thresholding

First, we apply a threshold to separate signal from background. The detected objects *(o)* are regions of connected pixels above the threshold (Fig 1C) in the image *g*. The pattern *O* of the threshold detected objects on the support *S*, with *S* representing the background of the image *g*, is:

$$O=\sum_{j=1}^{M}o_{j}\;with\;o_{j}\;\in \;S,\;\forall \;j\;\in \{1,2,3\ldots M\},$$ (4)

and *O* being a subset of *P*:

O⊆P. (5)

For the thresholding step, the images are initially filtered to smooth the signal, preserving object boundaries and supporting image binarization (Materials and Methods). If more than the 25% of the image pixels are above the threshold, we applied a watershed step to separate objects that touch each other and to aid the consequent segmentation. An automated threshold, implemented in ImageJ/Fiji, was chosen to compare sets of images (see Materials and Methods; for recommendations on threshold selection, see S1 Text).

#### 2. Tessellation

Next, tessellation is used to divide up the space occupied by the threshold-detected objects *(o)* into polygons, or tiles, based on the relationship of each object with its neighbors (Fig 1D). We implemented a procedure to take the extension of the objects into account. Given two neighboring objects (*U* and *V*), we measure the half way Euclidean distance ($\bar{uv}/2$) between *U* and *V* starting from their borders (*u* and v). To this aim, we used the skeletonization function first described by Lantuéjoul in 1977 [28]. This function removes pixels surrounding the threshold-detected objects until the tile boundaries between the objects remain (Fig 1D). This will lead to a number of tiles *T* that are similar to the number of objects (or watershed separated objects). Creating the tiles from object borders (*u* and v) is better suited than the common Voronoi tessellation [29], or watershed based approaches [30] (S2 Fig), to segment (pixel based) objects with different sizes and asymmetric shapes given that tiles are generated starting from the borders of the objects and not from their central point. For separated point sets, like in super-resolution images, the different strategies however converge.

#### 3. Tile area intensity measurement

As indicated in eq (3), the imaging process of the microscope leads to the generation of a blurred image. Therefore, depending on the resolution of the microscope, the threshold-detected object, *o* (eq (4)), in a tile may consist of multiple, spatially indistinguishable fluorescent emitters (in the context of eq (2), a number of *pf*). In Fig 1, for instance, the threshold-detected objects in tiles 2 and 5 are the result of blurring two emitters (simulated as single points), while the object in tile 4 is the result of blurring three emitters (see Fig 1A and 1D). We assume that the pixels of a tile *T* contain on average the intensity of the imaged fluorophores:

$$I_{T}≅P_{T}f,$$ (6)

where *I*_*T*_ represents the intensity of the tile *T*, containing a number *P*_*T*_ of single emitters, that are equally fluorescent, with brightness *f*. To determine the intensity of single tiles, the full set of tiles, obtained by skeletonization of the thresholded image (Fig 1D), is applied as regions of interest (ROIs) to the original smoothed grayscale image (Fig 1E). The intensity of each tile is then measured together with its area.

#### 4. Tile area correction

Since the tile intensity *I*_*T*_ varies with the number of fluorophores contained per tile, we aim to correct the area of brighter tiles based on their intensity by introducing a correction factor *C*. This correction factor represents the intensity of a fluorescent entity in the image (e.g., one fluorophore, or a group of fluorophores, depending on the resolution and sensitivity of the device). The correction is carried out by symmetrically dividing the tiles into multiple areas using the correction factor. Considering a tile of area *A*_*T*_ and intensity *I*_*T*_ and the correction factor *C*, with *C* being ∀ *C* ∈ ℝ|*C* > 0, if *I*_*T*_ > *C*, then the area *A*_*T*_ is corrected as *n* areas with the size of *A* according to:

$$A=A_{T}/n,$$ (7)

where *n* = [*I*_*T*_/*C*], with [] representing the greatest integer function. With this, the total area remains the same, as:

$$A_{T}=\sum_{k=1}^{n}A_{k}\text{ with }k\text{ being }\forall \;k\;\in \;\{1,2,3\ldots n\}.$$ (8)

If *I*_*T*_ < *C*, then *A* = *A*_*T*_, meaning no correction is applied (Fig 1F). Using this correction and if the correction factor equals the intensity of a single emitter (*C = f*), the number of tile areas obtained after correction would approximate the number of points *p*_*i*_ of the process *P*. For *C* larger than *f*, the analysis holds a relative distribution measure of multiples of *p*. As such the brightness information of a fluorescent component (i.e., a fluorophore) can be determined in a separate experiment offering the best correction, similar to Number and Brightness (N&B) approaches [31]. Alternatively, we implemented an estimation for the correction factor from the image itself. This is based on the distribution of tile intensities and yields a correction with the detectable intensity of a fluorescent entity from the image, as determined by the sensitivity of the microscope. A comprehensive description of correction factor determination can be found in S1 Text. An advantage of using the correction factor is the enrichment of tile area datasets with information otherwise ignored regarding the density of fluorophores contained within each tile. Incorporating intensity information into the distribution analysis makes QuASIMoDOH a powerful tool and a specific case in the analysis of marked point processes (processes characterized by a certain distribution and by a mark, which is, in this case, the intensity) [32].

#### 5. Tile area distribution analysis

The distribution of the obtained (corrected) tile areas is then modeled by the Inverse Gamma PDF [33] (Fig 1G). The Inverse Gamma was chosen based on literature [34,35] and on scoring several PDFs from a set of test distributions (see S1 Text and S1 Table). The Inverse Gamma is a continuous two-parameter PDF, defined over a support *x* > 0, as:

$$H(x;\alpha ,\beta )=\frac{\beta^{\alpha }}{\Gamma (\alpha )}x^{-\alpha -1}\text{exp}(-\frac{\beta }{x})$$ (9)

with shape parameter α and scale parameter β, and Г denoting the Gamma function. We used the Maximum Likelihood Estimation (MLE) of the Inverse Gamma parameters to identify values for the shape and scale parameters, which are descriptive of the point pattern. Once estimated, the parameters are used to fit tile area histograms for quality control of the MLE by calculation of the coefficient of determination r^2^. Fits with a quality less than r^2^ = 0.45 were discarded.

We next validated QuASIMoDOH using *in silico* generated images simulating various microscopy techniques and images of proteins and lipids with distinctly known distributions.

### Validation of QuASIMoDOH analysis principle using simulated microscopy images

**Fig 2:**
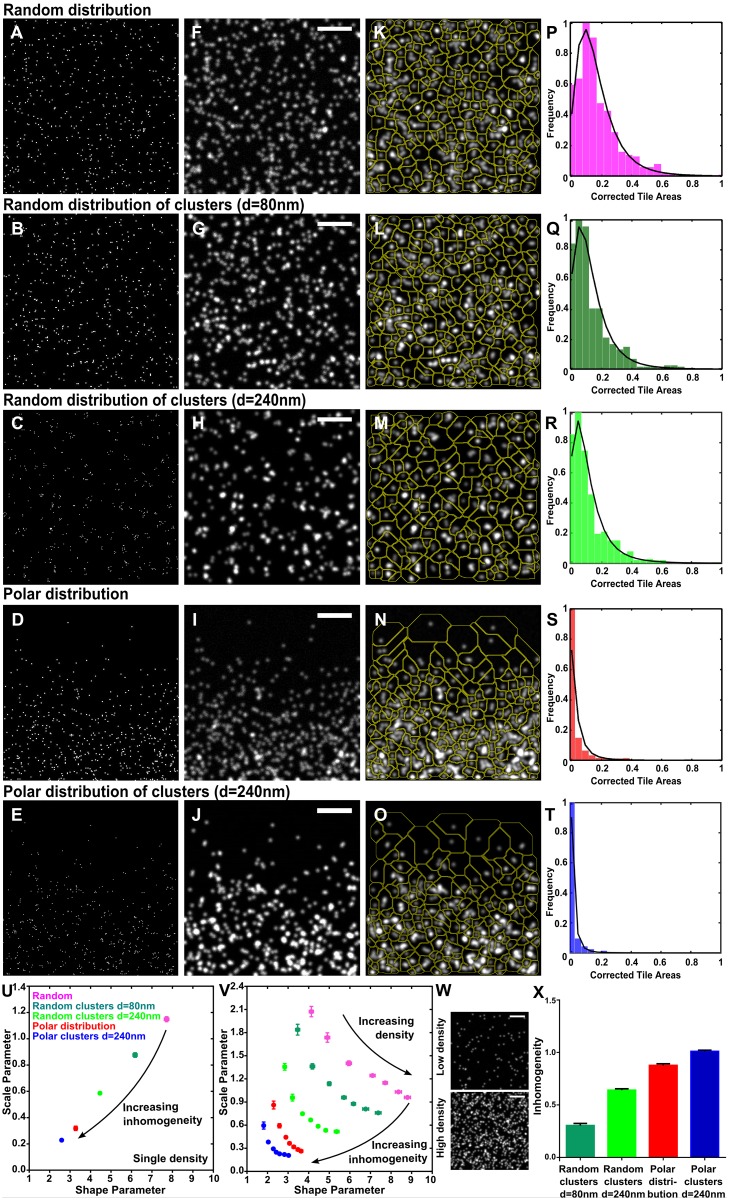
Basic principle of QuASIMoDOH analysis of point patterns. (A-E) Computer generated images of 500 points organized in (A) random distribution, (B) random distribution of clusters with diameter d = 80 nm, (C) random distribution of clusters with diameter d = 240 nm, (D) polarized distribution and (E) polarized distribution of clusters with diameter d = 240 nm. (F-J) Simulation of WF microscopy acquisition process on the point pattern images in (A-E). (K-O) Tessellation of the images in (F-J). (P-T) Histograms of the corrected tile areas fitted with the Inverse Gamma probability density function. (U) Plot of the two parameters from the Inverse Gamma PDF. Each point represents the average (and SEM) of 50 simulated WF images for each pattern, with the same density ρ ~5 (tiles/μm^2^). (V) Dependency of the shape and scale parameters from the inhomogeneity of the pattern and density. Each point represents the average (and SEM) of 50 simulated WF images for each pattern, with density 1 ≤ ρ ≤7 (tiles/μm^2^). (W) Two examples of images of random patterns with densities ρ ~1 (tiles/μm^2^) (top) and ρ ~7 (tiles/μm^2^) (bottom). (X) Inhomogeneity measure showing increasing inhomogeneity from clusters to polar patterns. Scale bar in (F-J, V): 2 μm.

To verify the ability of QuASIMoDOH to detect different distributions, we generated *in silico* images of different point patterns (see **‘Creation of discrete point pattern images’** in S1 Text and S3 Fig for a detailed description of the patterns). As described before, blur and noise were added to simulate the acquisition process of a fluorescence microscope (S1 Text, S1 Fig). We simulated images from widefield (WF) and Total Internal Reflection Fluorescence (TIRF) microscopy (resolution ~200 nm), Structured Illumination Microscopy (SIM) (resolution ~100 nm), and PALM (resolution ~20 nm). We decided to generate biologically relevant patterns, namely: random distributions (Fig 2A); random distributions of clusters with diameter d = 80 nm (Fig 2B); random distributions of clusters with diameter d = 240 nm (Fig 2C); polar distributions (Fig 2D); and polar distributions of clusters with diameter d = 240 nm (Fig 2E) for WF (Fig 2F–2J) and SIM images. For PALM, clusters with increasing sizes were simulated (see S1 and S3 Figs and S1 Text).

The correction factor used in the analysis of simulated images was determined by maximizing the accuracy of the analysis to give the correct number of known points. The accuracy is defined as the ratio between the number of areas, obtained by tile size correction, and the total number of points in the image (see S1 Text and S4 Fig). When the tessellation (Fig 2K–2O) and intensity correction are applied to images of different point patterns, the result is a set of tile areas typical for each pattern, captured by the histograms of corrected tile areas (Fig 2P–2T). The histograms display an increasingly steep peak for increasingly inhomogeneous patterns, moving from random to polar clusters. Modeling the distribution of tile areas by the Inverse Gamma PDF allows for the discrimination between different point patterns. The two parameters of the function are specifically associated with the histogram fit shape and its broadness. These parameters ultimately vary based on the underlying point pattern. In particular, they decrease with an increase in the inhomogeneity of the distribution, as shown in Fig 2U where the shape and scale parameters are plotted together.

### Density dependence

The average (and standard error of the mean, SEM) from the analysis of 50 images for each point pattern is presented in Fig 2U. The analyzed images have the same density, *ρ*, defined as:

$$\rho =N_{A}/\text{Are}{\text{a}}_{\text{Image}},$$ (10)

where *N*_*A*_ is the number of areas obtained upon tile size correction and Area_Image_ is the total area of the image in μm^2^. Fig 2U shows the results from analyzing images with density *ρ* ~5 (tiles/μm^2^); the images in Fig 2F–2J are examples of this density. Fig 2V shows a plot of the shape and scale parameters obtained by analyzing images with different patterns and densities (1 ≤ *ρ* ≤ 7 tiles/μm^2^). The value of the shape parameter increases with increasing density, while the scale parameter decreases. Examples of images of density *ρ* ~1 and *ρ* ~7 (tiles/μm^2^) are given in Fig 2W top and bottom, respectively. S5 Fig shows the densities associated with each point on the reference graphs for microscopy techniques with different resolution.

### Inhomogeneity measure

The deviation from a random distribution (calculated as the Euclidean distance from the random reference point) is used as a measure of the inhomogeneity of a point pattern. This is quantified in Fig 2X. Here the inhomogeneity is calculated for images with ρ ~5 (tiles/μm^2^) (Fig 2U). To provide comparable results for each density, the interval between the two extremes (random and polar clusters) was normalized to one. Analysis of images similar to the one shown in S6A Fig can result in shape and scale parameters larger than shape and scale parameters for the corresponding random distribution reference (S6B Fig). The inhomogeneity measure, in this case, will have a negative value (S6C Fig) since the coordinates of the random distribution are used as the origin from which the inhomogeneity is calculated. Shape and scale parameters 6–8 times larger than the random distribution reference can be further explained by a regular pattern where points are equally dispersed (S6D–S6F Fig).

Taken together, these results show that QuASIMoDOH can be used to quantify the inhomogeneity of spatial distributions and demonstrate the validity of the approach at the level of simulated images. We subsequently validated our method using images from fluorescence microscopy.

### Validation of QuASIMoDOH analysis principle by images of proteins and lipids with known distribution

Real and simulated images can exhibit analogous features, but they may differ as a consequence of microscope/detector sensitivity and dynamic range. Therefore, the correction factor *C* for actual microscopy images was selected to establish a comparable degree of correction as that determined by simulated results (see S1 Text and S7 Fig). We took advantage of plasma membrane sheets (S8 Fig) [36,37] to acquire images of the cell surface without fluorescent background arising from the cytoplasm [38,39]. To control for the integrity of the plasma membrane, cells were initially incubated with the lipophilic fluorescent stains DiO or DiI, depending on the combination of subsequent protein/lipid staining.

We tested whether QuASIMoDOH analysis was able to replicate findings made using microscopy techniques with different resolution and measured the inhomogeneity value from similar samples. We computed the cluster size of the lipid raft marker glycosylphosphatidylinositol (GPI)-anchored protein by comparing the results against the reference graph for simulated WF, SIM, and PALM images (S5 Fig). Images of GPI-anchored protein tagged with green fluorescent protein (GFP) or photoactivatable GFP (paGFP) [40], were acquired by WF, SIM, and PALM (S9A–S9C Fig). QuASIMoDOH analysis of SIM and WF data gave comparable results (S9D and S9E Fig). The analysis of 16 PALM images resulted in a GPI cluster size between 50 and 100 nm in diameter (two regions were identified as random and one region with a low r^2^ was excluded) (S9F Fig). PC-PALM analysis of the same 16 images resulted in an average GPI cluster size of approximately 80 nm in diameter (one region was identified as random). Obtained values for GPI cluster size were also consistent with previously published work [41,42]. Thus, QuASIMoDOH is capable of providing fast and quantitative analysis of super-resolution data with respect to cluster size. Finally, GPI inhomogeneity measurements from WF, SIM, and PALM data provided similar results when using appropriate tile area intensity corrections (see S9G Fig), indicating the applicability of the approach across microscopy techniques with various resolutions.

**Fig 3:**
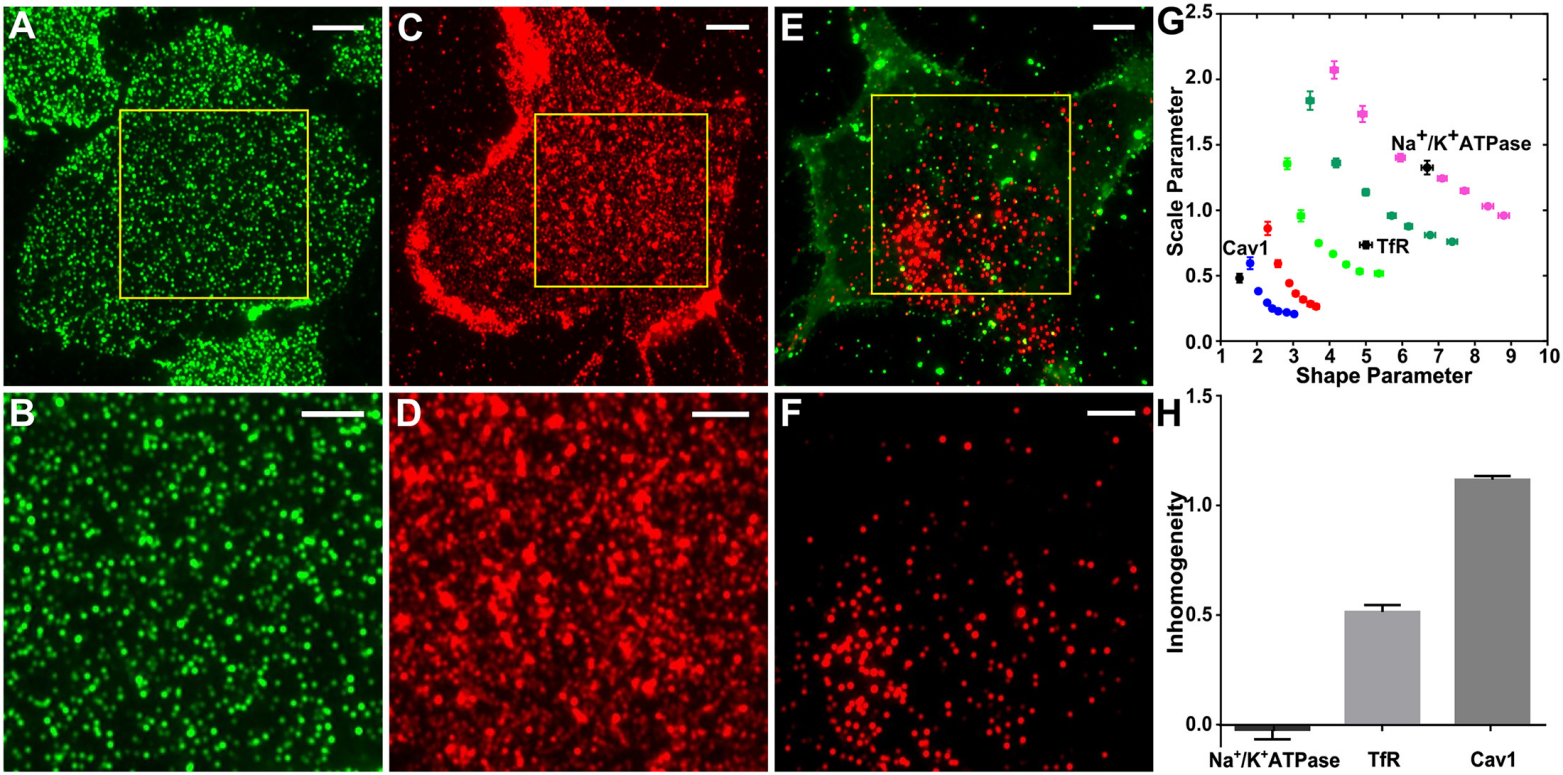
Detecting protein distributions using widefield microscopy. (A) Supported plasma membrane sheet of a MEF cell with Na^+^/K^+^ ATPase antibody staining. (B) Zoomed in region highlighted by the yellow rectangle in (A). (C) Plasma membrane sheet of a MEF cell exhibiting TfR antibody staining. (D) Zoomed in region highlighted by the yellow rectangle in (C). (E) Green: DiO staining for the MEF cell isolated plasma membrane. Red: Caveolin-1 antibody staining. (F) Zoomed in region highlighted by the yellow rectangle in (E). (G) The comparison with the analysis of simulated images suggests Na^+^/K^+^ ATPase was randomly distributed, TfR was organized into clusters, and Cav1 showed a polar distribution of clusters, as expected. (H) The inhomogeneity measure reveals a homogeneous distribution of Na^+^/K^+^ ATPase (deviation from random: -0.02) and increasing inhomogeneous organization for TfR (deviation from random: 0.51) and Cav1 (deviation from random: 1.1). Scale bars in (A, C, and E): 5 μm. Scale bars in (B, D, and F): 3 μm. Error bars represent the SEM. Number of analyzed images for Na^+^/K^+^ ATPase is 41, TfR is 47, and Cav1 is 50. The average r^2^ is 0.92, 0.93, and 0.93, respectively.

To further confirm that QuASIMoDOH detects different distributions we labeled three proteins that have well described and distinct spatial arrangements in Mouse Embryonic Fibroblasts (MEFs) using indirect immunostaining, and imaged these by WF microscopy. We used the sodium-potassium pump (Na^+^/K^+^ ATPase) [43] (Fig 3A and 3B), the transferrin receptor (TfR) [44] (Fig 3C and 3D), and caveolin1 (Cav1) [45] (Fig 3E and 3F). The Na^+^/K^+^ ATPase is an integral membrane protein that is randomly distributed across the cell surface. The TfR is an integral membrane protein responsible for ferric ion uptake, which clusters in clathrin-coated pits (200 nm in size) after ligand binding and prior to endocytosis. Cav-1 is a cytosolic peripheral-membrane protein that functions, together with cavin, in the formation of caveolae. Cav-1 is predominantly localized to the trailing edge of migrating cells, and thus shows a polarized distribution. Analysis by QuASIMoDOH demonstrates that the different distribution patterns of immunolocalized Na^+^/K^+^ ATPase, TfR, and Cav-1 can be reliably distinguished based on their inhomogeneity (Fig 3G and 3H). Comparing the results to simulated images indicates that the distribution pattern of Na^+^/K^+^ ATPase most closely resembles simulated images with a random distribution, whereas TfR matches those with a clustered distribution, and Cav1 aligns with simulated images highlighting a polar distribution of clusters. Thus, QuASIMoDOH effectively captures different protein distributions. Furthermore, these results demonstrate that our analysis can be successfully applied to images of proteins detected by immunolabeling.

**Fig 4:**
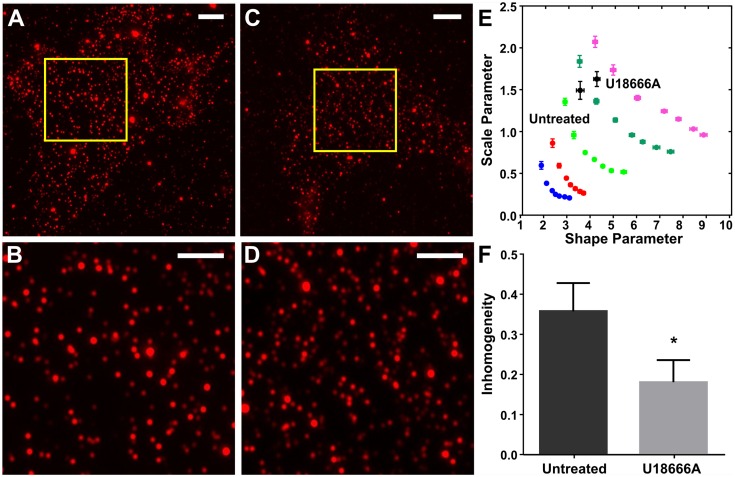
QuASIMoDOH reveals changes in lipid distribution patterns. (A) MEF cells stained for the lipid sphingomyelin using Lysenin. Images exhibit a characteristic clustered appearance of spots. (B) Zoomed in region indicated by the yellow rectangle in (A). (C) MEF cells were treated with 3 μg/mL U18666A for 18 h. (D) Zoomed in region indicated by the yellow rectangle in (C). (E-F) Upon drug treatment, the sphingomyelin distribution changes: clusters on the cell membrane appear to be smaller and more homogenous. The inhomogeneity measure reveals a shift to a more homogeneous distribution of Lysenin upon drug treatment (deviation from random significantly shifts from 0.36 to 0.18). Scale bar in (A and C): 5 μm. Scale bar in (B and D): 2 μm. Error bars represent the SEM. Analysis based on 49 cells for both untreated and U18666A treated cells. The average r^2^ is 0.72 and 0.75 for untreated and drug-treated cells, respectively.

The cell surface is also patterned by the wide variety of lipid molecules present in the plasma membrane. We, therefore, explored whether QuASIMoDOH detects lipid distributions, including changes that result from perturbation of lipid trafficking (Fig 4A–4D). For this purpose, we treated MEF cells with U18666A, an amphipathic steroid that causes cholesterol and sphingomyelin accumulation in late endosomes and lysosomes [46,47] (3 μg/mL for 18 h). The treatment was followed by fixation and incubation with Lysenin (a toxin that specifically binds sphingomyelin in the membrane [48,49]) and immunostaining. We reasoned that blocking endosomal sphingomyelin trafficking would deplete this lipid from the cell surface where it is normally organized into clusters, possibly causing reorganization to a more homogeneous distribution. Indeed, QuASIMoDOH analysis detects a significant difference in sphingomyelin organization between drug-treated and control cells (t-test: p = 0.045; two-tailed; unpaired, Fig 4E and 4F). Thus, QuASIMoDOH analysis can also detect plasma membrane lipid distributions and how these are altered in response to cellular perturbations.

**Fig 5:**
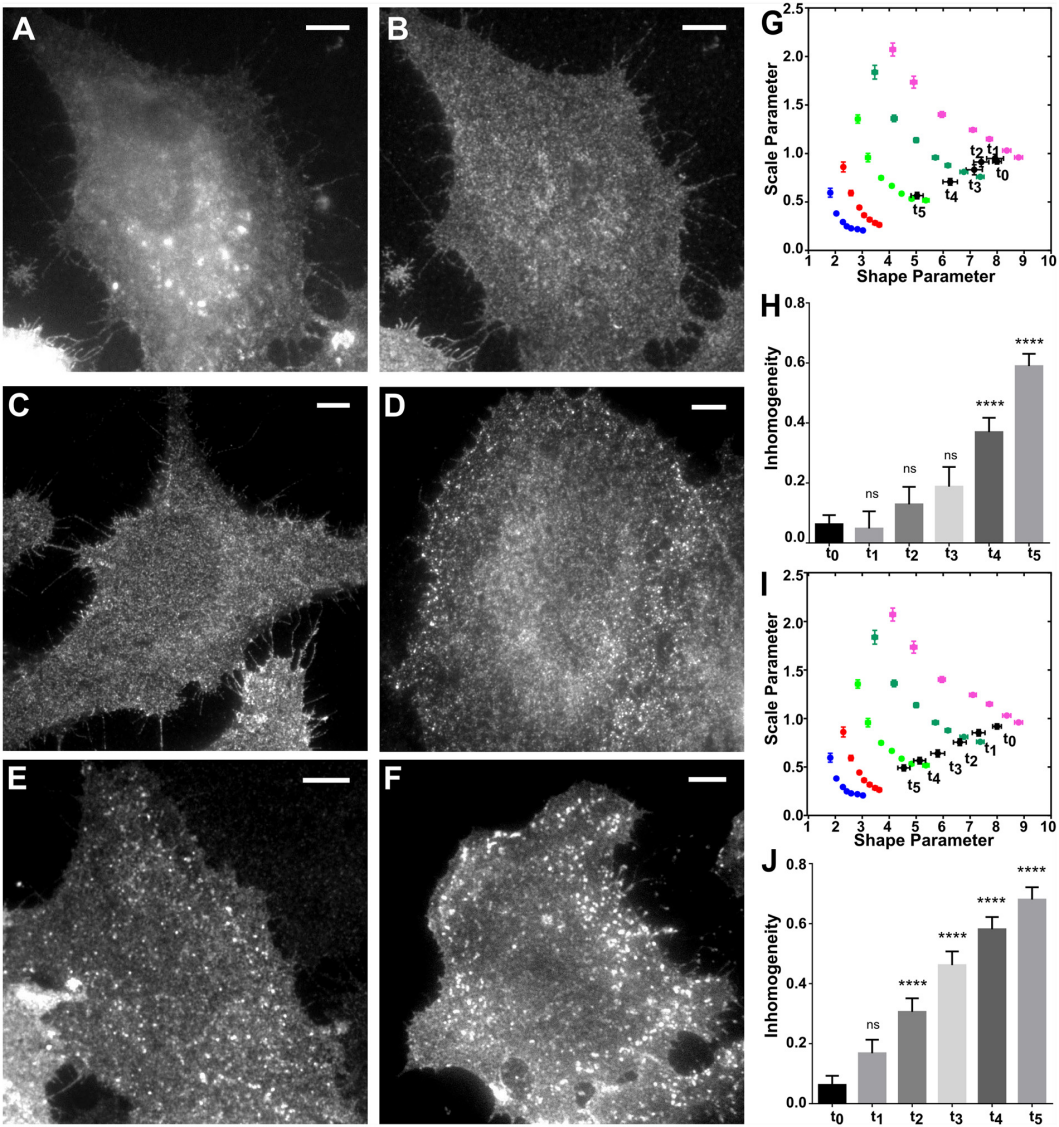
EGFR distribution analysis after EGF stimulation using TIRF imaging. HeLa cells were stimulated for different lengths of time with 2 ng/mL or 20 ng/mL EGF (t_1_ = 2 min, t_2_ = 5 min, t_3_ = 7 min, t_4_ = 10 min, t_5_ = 15 min) at 37°C, then fixed and imaged. (A and B) Distribution of EGFR before stimulation (t_0_), image acquired in (A) WF and (B) TIRF mode. (C-F) TIRF images acquired after 5 min of stimulation with (C) 2 ng/mL and (D) 20 ng/mL of EGF, and after 10 min of stimulation with (E) 2 ng/mL and (F) 20 ng/mL of EGF. The images show, over the time course of stimulation, that the EGFR becomes more clustered, likely through recruitment to endosomal structures. (G and H) Results from QuASIMoDOH analysis obtained after cell treatment with 2 ng/mL EGF. The time course (G) shows a successive deviation from a random distribution (t_0_) towards clusters resembling the reference point with a diameter of ~240 nm at t_5_. The inhomogeneity measure (H) increases with EGF incubation time. After 10 min, the change in the receptor distribution is significant (deviation from random for t_0_ and consecutive time points: 0.06, 0.05, 0.13, 0.19, 0.37, and 0.60). Results from one-way ANOVA, Dunnett’s multiple comparisons tests for t_0_ against all consecutive timepoints: p = 0.99, p = 0.75, p = 0.18, p<0.0001, and p<0.0001. (I and J) Results from QuASIMoDOH analysis obtained after treatment with 20 ng/mL EGF. In this case, the internalization rate of change is faster as inhomogeneities that resemble clusters with diameter d = 240 nm are present at the cell surface starting from t_4_ (I). The inhomogeneity measure (J) shows that the EGFR distribution at t_2_ is already significantly different from t_0_ (deviation from random for t_0_ and consecutive time points: 0.06, 0.17, 0.31, 0.46, 0.58, and 0.68). Results from one-way ANOVA, Dunnett’s multiple comparison test for t_0_ against all consecutive timepoints: p = 0.17, p = 0.0002, p<0.0001, p<0.0001, and p<0.0001. Scale bar: 5 μm. Error bars represent the SEM. At least 19 images were analyzed per time point. The average r^2^ in (G) is 0.87, 0.86, 0.86, 0.85, 0.84, and 0.81, for t_0_, t_1_, t_2_, t_3_, t_4_, and t_5_, respectively. The average r^2^ in (I) is 0.87, 0.86, 0.87, 0.83, 0.80, and 0.78, for t_0_, t_1_, t_2_, t_3_, t_4_, and t_5_, respectively.

To further explore QuASIMoDOH analysis in combination with different techniques we turned to TIRF imaging (with widefield resolution). We monitored the internalization of the epidermal growth factor receptor (EGFR) [50] (S10 Fig). We performed this in HeLa cells transiently transfected with EGFR-GFP, and treated with two concentrations of EGF reported to direct EGFR internalization distinctly [51,52]: a low EGF dose (2 ng/ml) induces EGFR internalization via the clathrin-mediated route, while a high EGF dose (20 ng/ml) directs EGFR internalization through both clathrin-coated pits and caveolae. TIRF images of cells fixed at specific time points (t_0_, before stimulation, t_1_ = 2, t_2_ = 5, t_3_ = 7, t_4_ = 10, and t_5_ = 15 minutes following EGF addition) were acquired, and the organization of EGFR at the plasma membrane was analyzed by QuASIMoDOH (Fig 5). This revealed that EGF indeed altered the EGFR distribution (Fig 5G–5J), corresponding to an increase in the measure of inhomogeneity. Furthermore, as predicted, this occurred more rapidly for cells exposed to the high concentration of EGF. These results demonstrate that QuASIMoDOH can be used to follow dynamic processes occurring at the cell surface and distinguish those processes with different rates.

### From global to local analysis

**Fig 6:**
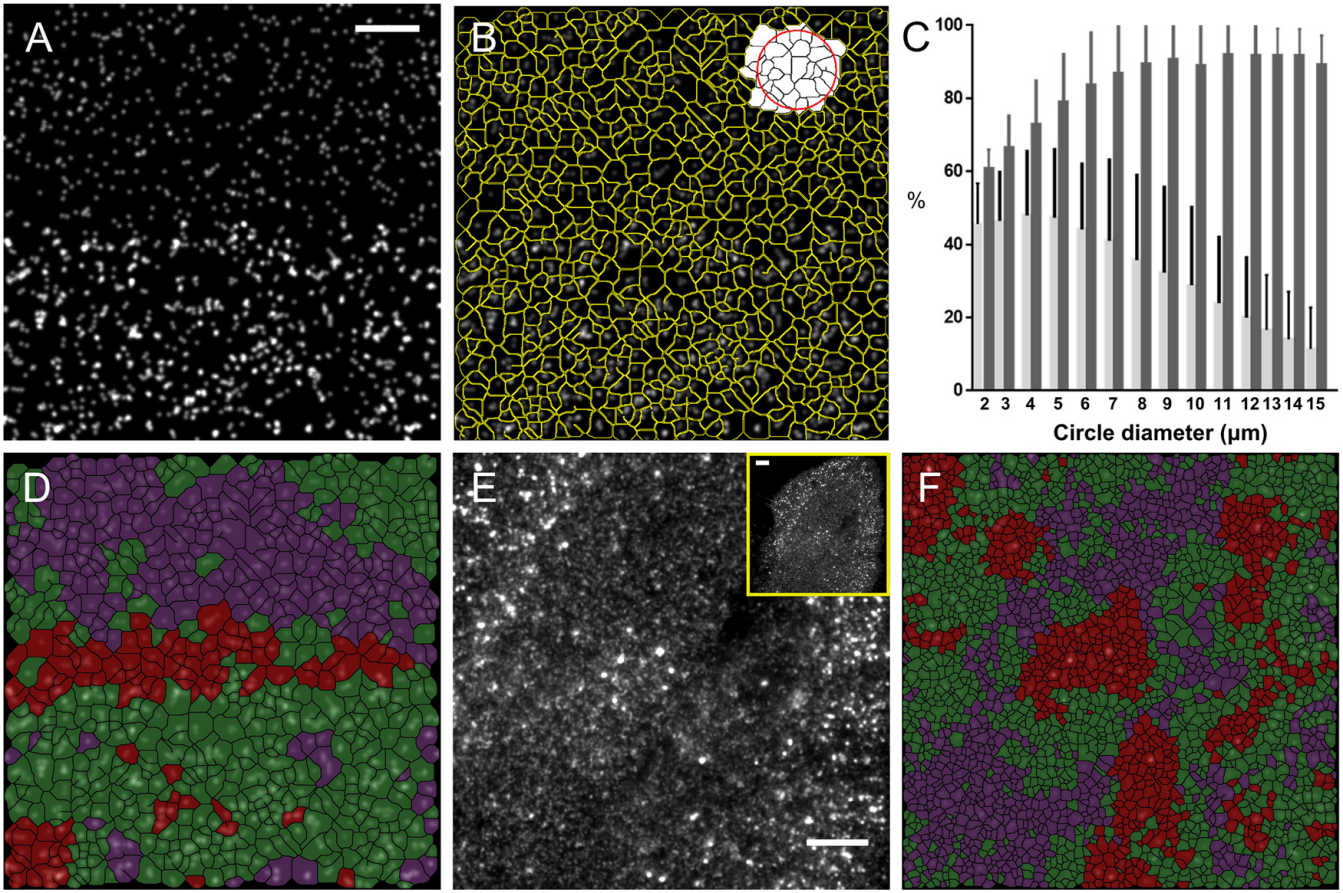
Local analysis of the EGFR internalization process. (A) Simulated image composed of random points (upper half) and clusters (lower half). (B) Example of local area (white tiles) selected by the red circle. (C) Plot of the percentage of tiles correctly detected as random in the upper third of 10 images generated as in (A) (light gray bar) and correctly detected as clusters in the lower third (dark gray bar). Error bars represent the standard deviation. (D) Local analysis of the image in (A) for diameters ranging from 4 to 10 μm and a minimum r^2^ of 0.45. Tile colors represent the closest reference point, with magenta for random, green for clusters (80 and 240 nm combined), and red representing polarized and polarized clusters (see reference graphs in Fig 2U and 2V). Non-colored areas are tiles with no assigned distribution. (E) EGFR image of a 10 min time point and EGF concentration of 20 ng/mL showing different organizations of EGFR on the surface: dispersed and clustered, potentially the result of localization to endosomal structures (see S10 Fig). (F) Local QuASIMoDOH analysis of (E). Scale bar in (A): 3 μm. Scale bars in (E): 5 μm.

The EGFR internalization assay raises the question: Can different spatial distributions observed in the plasma membrane (Fig 5E and 5F) be analyzed on a local scale as opposed to the global scale described above? To address this, we expanded QuASIMoDOH analysis to assess local distribution differences. For a local analysis, the tile area distribution analysis (see above ‘**5. Tile area distribution analysis**’) is applied to a subset of tiles in the images. This subset of tiles is selected by a circle that moves from the center of one tile to the next (Fig 6A and 6B). A color is assigned to the tile in the center based on the distribution detected using the selected tiles. Magenta represents a random distribution, green represents a distribution in clusters (for simplicity, the distinction between clusters of different sizes is omitted), and red represents polar distributions (for simplicity, the distinction between polar and polar clusters is omitted). When switching to a local analysis, the fitting quality (see S11 Fig) can be affected. To limit incorrect distribution assignment, we set 0.45 as a minimum r^2^ and a cut off for the outliers that, despite a high enough r², are too far away from any reference point (distance > 1, for widefield images), resulting in tiles without color.

To test the local analysis, we first created *in silico* images where the upper part contained a random distribution and the lower part consisted of a clustered distribution (see example in Fig 6A). These test images were simulations of WF images with a size of 512 x 512 pixels and 1600 points, on average. To maximize the number of tiles with detected distributions, the diameter of the circle must be in the range of 4 to 10 μm (Fig 6C, S1 Text and S12 Fig). As expected, from the local analysis we obtained a random distribution in the upper third of the image, a clustered distribution in the lowest third, and a polarized distribution in the center due to the neighborhood abrupt change from one part of the image to the other in this transition region (Fig 6D).

We next applied the local analysis to a fixed cell image from our EGFR internalization assay (treated for 10 min with high dose of EGF, Fig 6E). In Fig 6F, we applied the local analysis by setting the circle diameter in the range of 4 to 15 μm (similar to the simulation, the maximum diameter is about half of the image). A clear clustered organization of the receptor becomes apparent at the periphery of the cell following the analysis. A random distribution, however, covers the center of the cell and a gradient of random and clustered receptors mark the transition between these regions. Finally, an image of EGFR on the surface of a live cell, stimulated by 20 ng/mL of EGF, was used to further investigate the successful application of this local analysis approach. We were able to observe the local variation of receptor distributions following the time dependence of stimulation (see S1 Movie). These results indicate that QuASIMoDOH can be used to assess both the global and local changes in the distribution of fluorescent patterns at the plasma membrane.

## Discussion

Here we present QuASIMoDOH as a new approach to measure the inhomogeneity of a spatial distribution as a deviation from random towards clustered and polarized patterns (S13 Fig). Different from methods such as PC-PALM [20], Ripley’s K-function [14], nearest neighbor approaches [13], and DBSCAN [22,23], QuASIMoDOH is compatible with polarized distributions (see Caveolin-1 in Fig 3). It can detect and measure polarized distributions independent from their orientation, while other tools must acquire information on cell morphology or other features to assess the spatial phenotype of polarized molecules [53]. Compared to grid-based algorithms, like the Hoshen-Kopelman algorithm [54] that divides space into a grid and identifies clusters as continuously occupied areas, QuASIMoDOH can correct for unresolved points by complementing the tile area dataset with the information on tile intensity and is thus applicable beyond single molecule imaging techniques.

Where SpIDA [55] measures protein interactions and aggregation by multiple fitting of pixel intensity histograms, QuASIMoDOH analyzes the fit from tile intensity histograms to extract a measure of plasma membrane inhomogeneity. Comparable to Number and Brightness (N&B) approaches [31], which analyze temporal fluctuations, in QuASIMoDOH the tile intensity *I*_*T*_ depends on the number of molecules (*p*) in the tile and their brightness (*f*): *I*_*T*_ = *pf*. After calibration, incorporating background intensity, N&B can be mapped to absolute values with good spatial resolution. For ease of use in QuASIMoDOH, we decided to estimate the correction factor from the image by an analysis of the distribution of tile intensities. Determining the correction factor, however, leaves the possibility of misinterpretation due to background, non-uniform illumination, or the presence of artifacts. Pre-processing steps to address these conditions are provided in the QuASIMoDOH documentation (S1 File). S14 Fig offers information on how to determine if an image is suitable for QuASIMoDOH analysis.

We applied QuASIMoDOH to PALM, SIM, WF, and TIRF images (Figs 3–5, S9 Fig). However, in principle, this tool can also be applied to other microscopy modalities, including confocal microscopy and (d)STORM imaging. We have demonstrated that results obtained for the cluster analysis of GPI-anchored proteins are in reasonable agreement across microscopy techniques with different resolution and with previously published results obtained by PC-PALM analysis [42]. Additionally, QuASIMoDOH correctly detected the differential distribution of Na^+^/K^+^ ATPase, TfR, and Cav-1 proteins. Notably, QuASIMoDOH does not require the presence of a reference molecule in the sample with a known distribution. As a result, comparisons between protein/lipid organization in different cell types or within the same cell and under different experimental conditions are possible, e.g., evaluating changes in a lipid distribution following drug treatment. We specifically demonstrated this by analyzing the distribution of sphingomyelin (Fig 4). The observed increase in homogeneity for the lipid probe Lysenin in cells treated with U18666A compared to untreated cells emphasizes the potential of our analysis tool to readily test a range of cell perturbations. Additionally, the fact that affinity probes, fusion proteins, and lipid probes can be detected highlights the versatility of our analysis method. We further demonstrated the ability of this approach to reveal the dynamic nature of EGFR organization at the plasma membrane upon stimulation, using both fixed and live cells.

A list of advantages and limitations of QuASIMoDOH is provided in Table 1. An important feature of QuASIMoDOH analysis is its direct application to microscopy data with no detailed prior knowledge of the sample. Overall QuASIMoDOH serves as a quick, straightforward, and automatable method to measure distribution patterns of proteins and lipids on the cell surface. It can be used to study events at the cell surface related to cell signaling or remodeling as these appear, for example, in the reprogramming of cancer cells or neuronal differentiation.

**Table 1:** Advantages and limitations of QuASIMoDOH.

Advantages	Limitations
Able to detect, aside from random and clustered distributions, polarized patterns without knowledge of polarity axis.	The analysis provides the closest inhomogeneity reference point that resembles the distribution of given data or a relative inhomogeneity measure.
Input to estimate cluster shapes, densities, or numbers is not required.	The number of molecules per cluster is not calculated.
Plugin optimized for images acquired by microscopy techniques with different resolution.	Although the specific PDF used for fitting the data is based on earlier publications [34,35,56], and on scoring multiple PDFs (S1 Text), other PDFs may be better suited for particular conditions.
A reference marker in the sample is not required. Thus, it is possible to assess differences in the organization of plasma membrane components independently from cell type, drug treatments, and membrane perturbations.	
Both protein and lipid patterns can be analyzed independently from the labeling method.	Inhomogeneity reference point graphs are based on parameters used in simulated images. Thus, more acute pattern gradients may be outside the graph limits, or extended clusters may mimic polar distributions.
Rapid screening of images with limited pre-processing.	
“Global analysis” assesses the distribution of fluorescence across large areas of the cell. “Localized analysis” allows detection of local variations in the distribution of spatial patterns.	
Rapid computation of the analysis.	The statistical power of histogram fitting decreases if too few objects are sampled (S14 Fig).
A ImageJ/Fiji plugin is available for download (S1 File, S13 Fig).	

## Materials and Methods

### Data processing

For QuASIMoDOH development and testing, a Dell Optiplex7010 computer was used (Intel CITM) i7-3770 CPU @ 3.40GHz processor and 4.00 GB RAM, running Windows 7 Professional). Images were run in batch mode, each 256 x 256 pixels in size. In total, approximately 25,000 points were identified, and using a standard four year old personal computer, we were able to analyze the entire dataset of 50 images in 22 seconds. Pixel-by-pixel image processing of both simulated and fluorescent images was carried out using custom-made routines in MATLAB and ImageJ/Fiji [26] according to the schemes described in the main text. For plasma sheet samples, background correction was applied (where necessary) by subtracting a background value either using the Dip Image function “backgroundoffset” or manually. Background subtraction on TIRF images of intact cells was carried out using the ImageJ/Fiji function “Rolling Ball” [26].

The WF, SIM, and TIRF images are initially filtered by the ImageJ/Fiji filter ‘Sigma Filter Plus’ and then smoothed. For the separation of signal and background in our simulated images, we used the default threshold in ImageJ/Fiji [57], which is based on Isodata. For the widefield images of Na^+^/K^+^ ATPase and Caveolin-1, the threshold Li [58] was used. GPI, TfR, and Lysenin widefield images, as well as GPI SIM and EGFR TIRF images, were thresholded using Mean [59]. Apparently, the best-suited threshold for an image can depend on the imaging modality and was chosen upfront based on the obtained images (see S1 Text). For skeletonization, the function implemented in ImageJ/Fiji version 1.47 was used. PALM super-resolution images were generated by analyzing datasets obtained from Peak Selector software (Research Systems, Inc.). Analysis of paGFP-GPI images was performed on 16 square regions of 7–18 μm^2^ obtained from 8 cells, with an average of 75 ± 5 localized peaks/μm^2^ and average localization precision of 15 nm. PC-PALM analysis was performed similarly to the previously described method [20]. For QuASIMoDOH analysis, localized peaks were grouped using a group radius of 3 x maximum localization precision and a maximum dark time of 5 s using Peak Selector. The maximum dark time was obtained experimentally using sparse paGFP. Similar to the method previously reported by Annibale et al. [60], a best fit of the observed fluorophore counts as a function of the dark time was used to determine the effective number of molecules present in the sample. Grouped peak coordinates (in pixels) were subsequently fed into ImageJ/Fiji for QuASIMoDOH analysis. Peaks were plotted following a conversion to nanometers by a user-defined pixel size. The image width and height was calculated from the coordinates as the difference between the maximum and minimum values of X and Y coordinates, respectively. The generated image was then scaled to a 2.5 nm pixel size, and subsequently analyzed as described before.

All graphing and statistics were prepared using MATLAB, GraphPad Prism (GraphPad, La Jolla, USA), and Excel (Microsoft, Redmond, USA). For the analysis of distribution patterns of proteins and lipids, images of cells from three different preparations/coverslips were analyzed. Images were pooled for further QuASIMoDOH analysis.

### Cell culture

Mouse Embryonic Fibroblast (MEF) cells were maintained at 37°C and 5% CO_2_ in DMEM/F12 (+L-glutamine + 15 mM HEPES) supplemented with 10% of fetal bovine serum (FBS). MDA-MB-468 cells were cultured in DMEM supplemented with 10% FBS. Transient transfection of MDA-MB-468 cells was performed using Jetprime (PolyPlus, following manufacturer instructions) with 2 μg of paGFP-GPI similarly as described in detail elsewhere [42]. Transient transfection of MDA-MB-468 cells was performed using FuGENE (Promega, following manufacturer instructions) with 2 μg of GFP-GPI. HeLa cells were grown in DMEM/F12 (+L-glutamine + 15 mM HEPES) supplemented with 10% FBS. One day after plating cells, transfection was performed using FuGENE6 with 0.5 μM of plasmid pGFP encoding for EGFR-GFP.

### Supported plasma membrane sheets preparation

Plasma membrane sheets were prepared as previously described [36]. In short, MEF cells were cultured in DMEM/F12 (+L-glutamine + 15 mM HEPES) supplemented with 10% of fetal bovine serum. Cells were grown on coverslips to approximately 60% confluence. At 4°C, cells were washed with PBS^+/+^ and subsequently with coating buffer (20 mM MES, 135 mM NaCl, 0.5 mM CaCl_2_, 1 mM MgCl_2_, pH5.5). Next, they were incubated in coating buffer with 1% of silica beads for 30 min. They were rinsed with deionized water (10 min) followed by three washing steps with PBS^+/+^. To prepare plasma membrane sheets, shear force was applied to the coverslip using a syringe held at a 30° angle (on the coverslip). As the upper surface of adherent cells is made rigid by the silica coating, shear forces break off membranes at the edges releasing all soluble contents and retaining only the basal plasma membrane adherent to the coverslip. These remaining sheets were then fixed (4% paraformaldehyde in PBS, 15–20 min at RT) (see schematic in S8 Fig).

### Immunostaining

Fixed plasma membrane sheets were rinsed with PBS^+/+^ and blocked (2% FCS, 2% BSA, 0.2% gelatin, 5% goat serum in PBS^-/-^) for 1 h. For immunofluorescence, the following antibodies were used: mab to murine Cav-1 (BD Biosciences; San Jose, USA) (1:200); mab against Transferrin receptor (Zymed/Life Technologies) (1:100); mab against Na^+^/K^+^ ATPase alpha (Novus Biologicals, Littleton USA) (1:100). Alexa488/555/568 conjugated secondary antibodies were used (Life Technologies) (1:1000). Lysenin (1:40) was purchased from Peptide Institute (Osaka, Japan) and immunolocalized using anti-Lysenin pab (1:100) followed by goat anti-rabbit Alexa-555 (Life Technologies) secondary antibody. U18666A was purchased from Sigma. DiI and DiO (Life Technologies) (1:100) were used to stain lipid bilayers. Staining was performed according to manufacturer recommendations.

### EGFR stimulation

Twenty-four hours after transfection, HeLa cells were serum starved overnight. For the preparation of the fixed samples, after starvation, the cells were incubated at 37°C with either 2 ng/mL or 20 ng/mL EGF for different time intervals (2, 5, 7, 10, and 15 minutes), then fixed with 4% paraformaldehyde in PBS, for 15–20 minutes at room temperature. The fixed samples were then imaged by TIRF. Additionally, living cells were imaged by TIRF upon stimulation with 20 ng/mL of EGF (S1 Movie).

### Imaging

Widefield and structured illumination images were acquired with a structured illumination microscope (Elyra S1 (Carl Zeiss, Jena, Germany) equipped with a 63x oil objective lens with a numerical aperture (NA) of 1.4 and an Andor iXon 885 EM-CCD camera). PALM imaging was performed using a Nikon Instruments Ti Eclipse inverted microscope with a 100x/1.49 NA TIRF objective (Apo) and a 488 nm laser (Agilent, MLC-MBP-ND laser launch) with an EM-CCD camera (Andor Technology, iXon DU897-Ultra). The microscope was equipped with a Perfect Focus Motor to minimize axial drift over the duration of imaging. paGFP was simultaneously activated and excited with the 488 nm laser at an intensity set to 1.45–1.9 mW (as measured at the optical fiber). Exposure time was set at 100 ms. Imaging was performed until paGFP was completely exhausted, typically after 20,000 frames. TetraSpeck beads (Life Technologies) were used as fiducial markers for drift-correction during image acquisition. TIRF images were acquired by a Nikon Ti Eclipse inverted microscope, equipped with a 100x/1.49 NA oil objective lens and a Hamamatsu Orca D2 camera, or Hamamatsu Orca Flash 4 Lite, for living cell imaging. Confocal images were acquired using the same microscope equipped with Nikon A1R using a 60x 1.4 NA oil objective. Co-localization analysis was carried out using the Fiji plugin JACoP [61].

## Supporting Information

S1 Fig Simulating the imaging process from a microscope. (A) Ideal image of 10 points (pixels) distributed in 2D space. (B) Blurred image obtained after simulating the Point Spread Function (PSF) using a Gaussian profile. (C) Photon noise is introduced in the image, leading to points with variable intensity. (D) Background noise is simulated by a Gaussian distribution. The final simulated image contains Poisson noise and Gaussian noise. Scale bar: 1 μm.
(TIF)

S2 Fig Comparison of tessellation techniques. (A) Binary image obtained by thresholding the image in S1D Fig. (B) Image result obtained from skeletonization. (C) Image result obtained by Voronoi tessellation as well as by watershed function.
(TIF)

S3 Fig Generation of point pattern images. Schematic overview of preparing point patterns. A multi-step process was established to provide images with different point distributions and levels of complexity.
(TIF)

S4 Fig Correction in simulated images. Simulations of random distribution with different resolution, (A) WF of 250 nm resolution, (B) WF of 200 nm, (C) structured illumination of 150 nm and (D) 100 nm. Scale bars: 2 μm. (E) Comparison of correction degree for images as in (A-D). 10 images each were analyzed, average and SEM are shown. (F) Normalized tile intensity distribution of (B). Intensity of tiles F1-3 is indicated in the graph. F1-3 are examples of tiles containing one, two and three points, taken from image in (B). Accuracy obtained for (G) simulated WF images and (H) simulated SIM images by running QuASIMoDOH analysis without tile area correction (No Correction), with correction using the minimum tile intensity (Minimum), the average of the 5% smallest values (Average 5%), the average of the 10% smallest values (Average 10%), and the first quartile (Quartile 1). The average (and SEM) of 175 images is displayed. Images with different pattern types and percentage of pixels above the threshold ranging from 5% to 35% were used. The dotted line is placed at 1 and represents 100% accuracy. Values below 1 indicate an underestimation of the number of points, values above 1 indicate an overestimation of the number of points.
(TIF)

S5 Fig Reference graph densities. (A) Reference graph for WF and TIRF images. (B) Reference graph for SIM images. (C) Reference graph for PALM images. The densities, *ρ* (tiles/μm^2^), of the simulated microscopy images are indicated in the graph.
(TIF)

S6 Fig Inhomogeneity measure. (A) Simulated WF image of a random pattern with density *ρ* = 5 (tiles/μm^2^). (B) Shape and scale parameters characteristic for images with density *ρ* = 5 (tiles/μm^2^) are plotted for the various point patterns (random, random clusters with diameter d = 80 nm, random clusters with diameter d = 240 nm, polar and polar clusters with diameter d = 240 nm). The white part of the graph represents the area where the deviation from random is calculated as a positive value. Results falling in the grey part of the graph, however, have negative values and underline the fact that the shape and scale parameters are larger than those for the reference of a random distribution. In this graph, the black point is the result from analysis on the image in (A). (C) The inhomogeneity measure increases from clustered to polar patterns. The black bar has a negative inhomogeneity value because the result from analysis on the image in (A) has shape and scale parameters larger than the random distribution reference point. (D) Plot of shape and scale parameters, including the results from analyzing images with regular patterns of density 1 ≤ *ρ* ≤ 7 (tiles/μm^2^) (black points, example images shown in E and F). Each point represents the average (and SEM) of 50 simulated WF images for each patter and density. (E) Simulated WF image of a regular pattern with density *ρ* = 1 (tiles/μm^2^). (F) Simulated WF image of a regular pattern with density *ρ* = 7 (tiles/μm^2^). Scale bar in (A, E, F): 2 μm.
(TIF)

S7 Fig Correction of real images of test samples. To imitate biological samples, test samples of cell surface receptor staining were prepared. (A) WF and (B) SIM images of randomly dispersed EGFR primary antibody stained with goat anti-mouse Alexa-488 on coverslips. Scale bars: 2 μm. (C) Normalized tile intensity distribution of (A). Intensity of tiles C1-3 is indicated in the graph. C1-3 are examples of tiles containing the detected single unit (C1), two and three units (C2 and C3, respectively) taken from the image in (A). (D, E) Correction degree. Images of WF (D) and SIM (E) were analyzed with QuASIMoDOH by using a correction factor of the 5% average of the lowest tile intensity values (Average 5%), the 10% of the lowest tile intensity values (Average 10%), the first quartile (Quartile 1), and the median (Median) of the tile intensity values. Black bar labeled ‘Simulations’ shows the comparison to simulated images (see as well S4 Fig). The average (and SEM) of 25 images is displayed (percentage of pixels above the threshold ranging from 5% to 25%). First quartile and median, for WF and SIM images, respectively, seem to provide the best guess for *C* from the image, given the same degree of correction as used in the simulations. (F, G) QuASIMoDOH analysis results of WF (F) and SIM (G) images obtained using first quartile and median, respectively, as correction factors.
(TIF)

S8 Fig Procedure for preparing supported plasma membrane sheets. (A) Cells are first seeded and grown on a coverslip. (B) Cells are subsequently incubated with silica beads to coat the apical membrane. (C) After hypotonic swelling, the coated apical membrane is then removed by applying shear force.
(TIF)

S9 Fig Application of QuASIMoDOH across microscopy techniques with different resolution. Analysis of the distribution of GPI on the surface of MDA-MB-468 cells. (A) WF and (B) SIM image of GPI-GFP on a supported plasma membrane sheet. (C) Rendered PALM image of paGFP-GPI. Analysis of the GPI distribution from (D) WF, (E) SIM, and (F) PALM images. (G) Comparison of the inhomogeneity measure. Normalization across densities was carried out between the reference point for a random pattern and clusters with diameter d = 240 nm. Scale bar in (A and B): 2 μm. Scale bar in (C): 500 nm (see Materials and Methods for image details). Error bars in (D-G) represent standard error of the mean. Analysis in (A and B) is based on 25 cells, analysis in (C) is based on 16 ROIs. The average r^2^ in (A) is 0.82, in (B) is 0.64, and in (C) is 0.74.
(TIF)

S10 Fig Confocal images of EGFR. The internalization of EGFR was followed using confocal imaging of HeLa cells (A) before incubation with EGF and (B-F) at 2, 5, 7, 10, and 15 minutes after incubation with 20 ng/ml of EGF. The top panels represent the distribution of EGFR-GFP (in green), the middle panels show the early endosome marker EEA1 (in red), and the bottom panels are images of the merged channels. (G) Quantification of colocalization between EGFR and early endosomes, using Manders’ coefficient. The graph shows an increased colocalization after two minutes of cell incubation with EGF. Scale bars (A-F): 5 μm.
(TIF)

S11 Fig Dependency of r^2^ values on the number of tiles as estimated from the inverse gamma parameters. The r^2^ value increases with an increase in the number of tiles. Analysis based on 60 images per point. Error bars represent SEM.
(TIF)

S12 Fig Local analysis of the EGFR internalization process. The local analysis results using different diameters (A) and different diameter intervals (B) are displayed for the image shown in Fig 6A.
(TIF)

S13 Fig QuASIMoDOH method overview. Left graph: analysis steps. Right graph: workflow.
(TIF)

S14 Fig Flowchart guide for QuASIMoDOH analysis applicability. (TIF)

S1 Table Tested probability density functions (PDFs) and relative coefficient of determination (r^2^). (PDF)

S1 Text Details of QuASIMoDOH analysis development. The supplementary text provides additional information about the creation of discrete point pattern images, simulating the imaging process, threshold selection, determination of the probability density function, determination of the correction factor, and local analysis: circle size determination.
(DOCX)

S1 Movie Local analysis of EGFR internalization over time. Sequence of TIRF images of EGFR-GFP on the surface of a HeLa cell incubated with EGF. (A) TIRF image of EGFR-GFP on the surface of a HeLa cell. (B) Zoomed in region of the area delimited by the yellow rectangle in (A). (C) Local analysis of the ROI shown in (B).
(AVI)

S1 File ImageJ/Fiji plugin and example data to test QuASIMoDOH. The folder contains (i) the plugin ‘Quasimodoh_Analysis-1.0.0.jar’ for running QuASIMoDOH using ImageJ/Fiji, (ii) the documentation of the plugin ‘QuASIMoDOH Analysis Documentation.pdf’ and (iii) different datasets in the folder ‘Test Data’. The proteins imaged are indicated.
(ZIP)
