# Supplementary figures and images for: Inhomogeneity Based Characterization of Distribution Patterns on the Plasma Membrane
Source: PLoS Comput Biol. 2016 Sep 7;12(9):e1005095. doi: 10.1371/journal.pcbi.1005095 (PMC5014321; doi:10.1371/journal.pcbi.1005095)

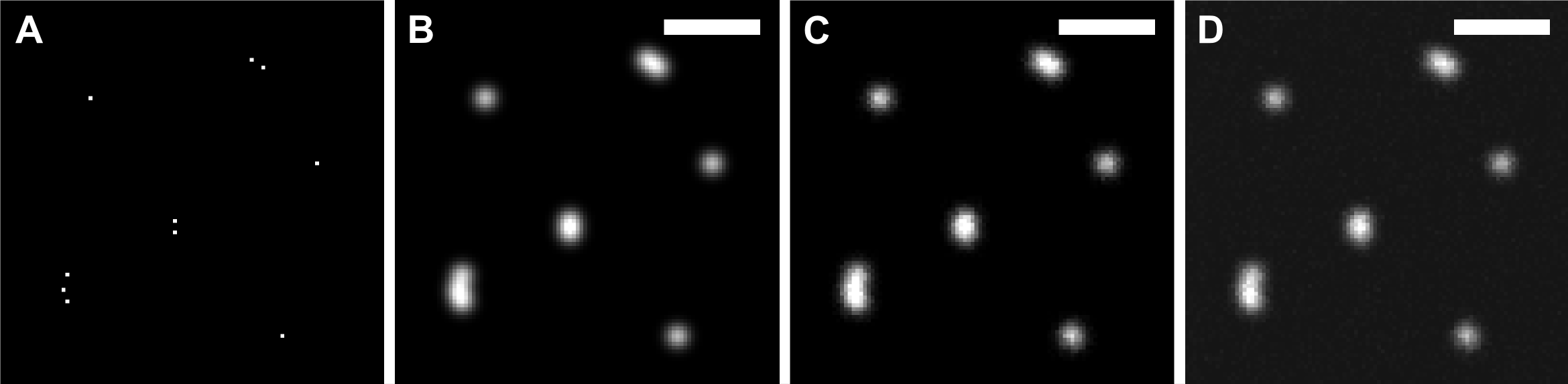

Supplement: S1 Fig — (A) Ideal image of 10 points (pixels) distributed in 2D space. (B) Blurred image obtained after simulating the Point Spread Function (PSF) using a Gaussian profile. (C) Photon noise is introduced in the image, leading to points with variable intensity. (D) Background noise is simulated by a Gaussian distribution. The final simulated image contains Poisson noise and Gaussian noise. Scale bar: 1 μm. (TIF) [file pcbi.1005095.s001.tif]

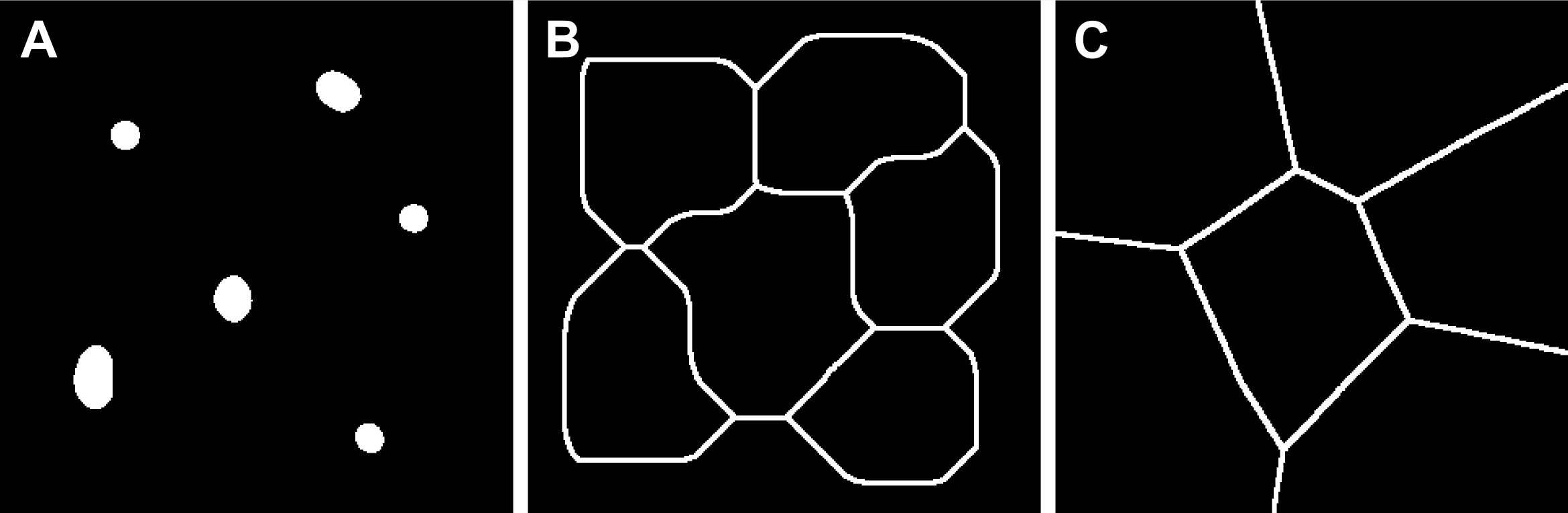

Supplement: S2 Fig — (A) Binary image obtained by thresholding the image in S1D Fig. (B) Image result obtained from skeletonization. (C) Image result obtained by Voronoi tessellation as well as by watershed function. (TIF) [file pcbi.1005095.s002.tif]

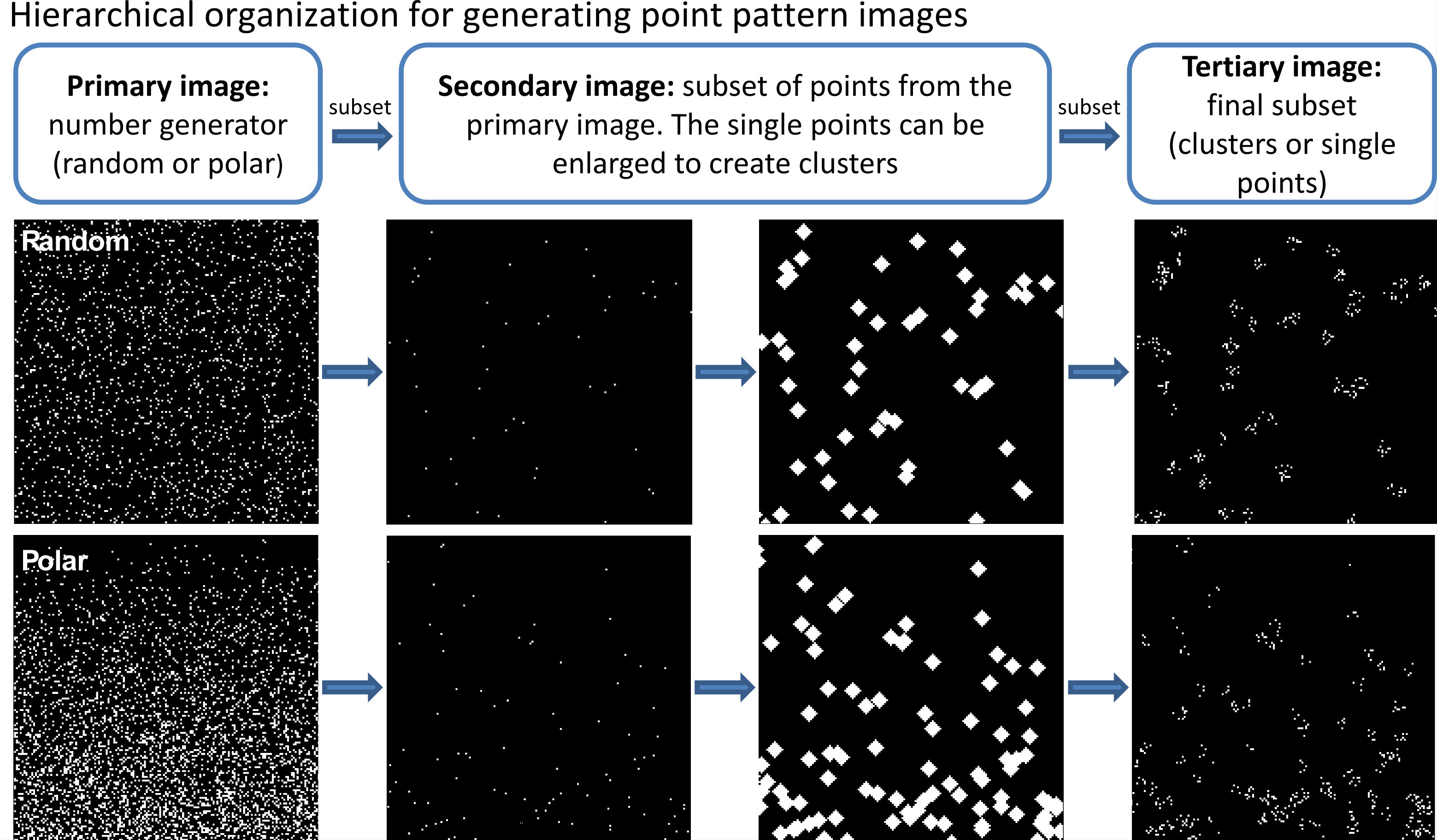

Supplement: S3 Fig — Schematic overview of preparing point patterns. A multi-step process was established to provide images with different point distributions and levels of complexity. (TIF) [file pcbi.1005095.s003.tif]

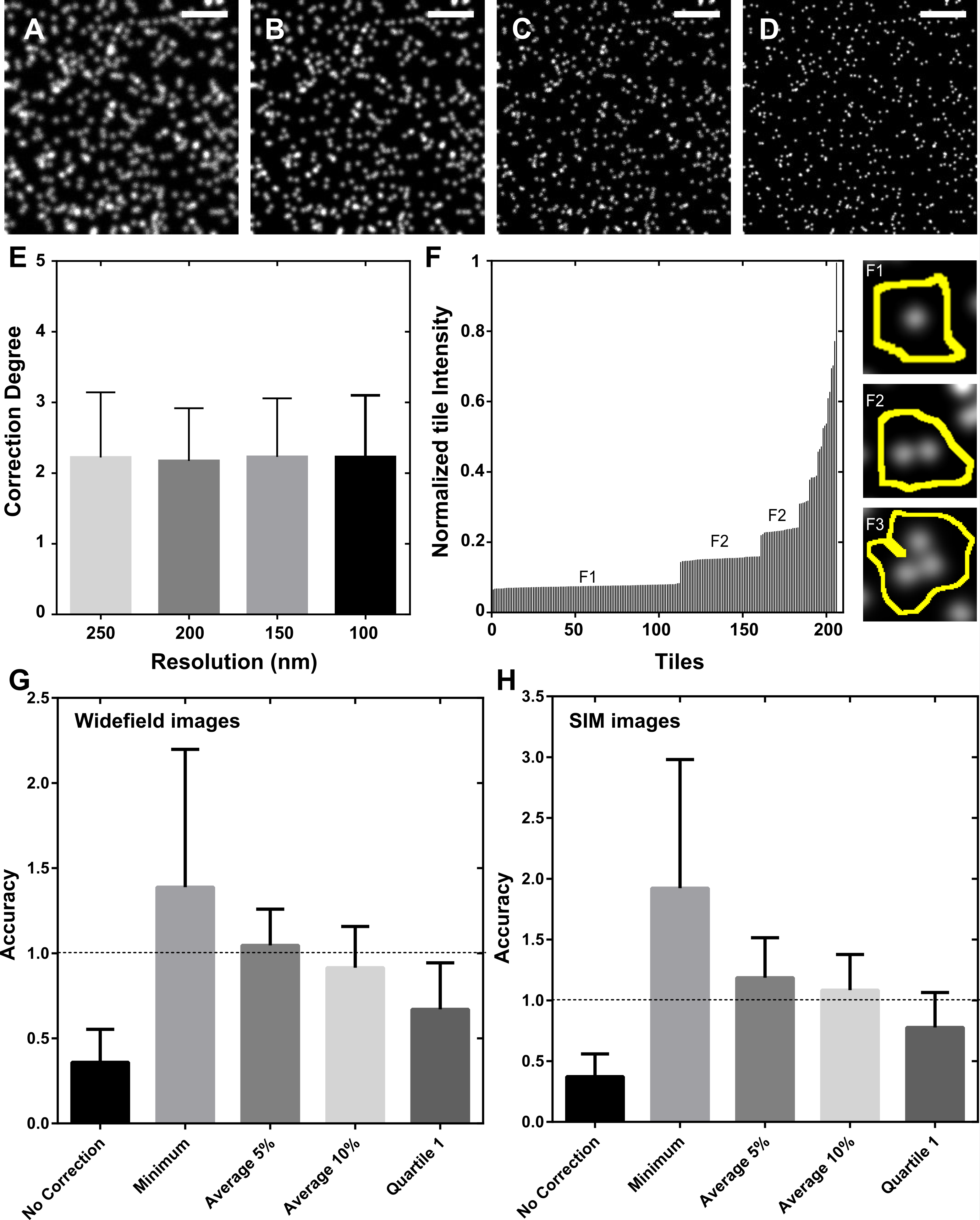

Supplement: S4 Fig — Simulations of random distribution with different resolution, (A) WF of 250 nm resolution, (B) WF of 200 nm, (C) structured illumination of 150 nm and (D) 100 nm. Scale bars: 2 μm. (E) Comparison of correction degree for images as in (A-D). 10 images each were analyzed, average and SEM are shown. (F) Normalized tile intensity distribution of (B). Intensity of tiles F1-3 is indicated in the graph. F1-3 are examples of tiles containing one, two and three points, taken from image in (B). Accuracy obtained for (G) simulated WF images and (H) simulated SIM images by running QuASIMoDOH analysis without tile area correction (No Correction), with correction using the minimum tile intensity (Minimum), the average of the 5% smallest values (Average 5%), the average of the 10% smallest values (Average 10%), and the first quartile (Quartile 1). The average (and SEM) of 175 images is displayed. Images with different pattern types and percentage of pixels above the threshold ranging from 5% to 35% were used. The dotted line is placed at 1 and represents 100% accuracy. Values below 1 indicate an underestimation of the number of points, values above 1 indicate an overestimation of the number of points. (TIF) [file pcbi.1005095.s004.tif]

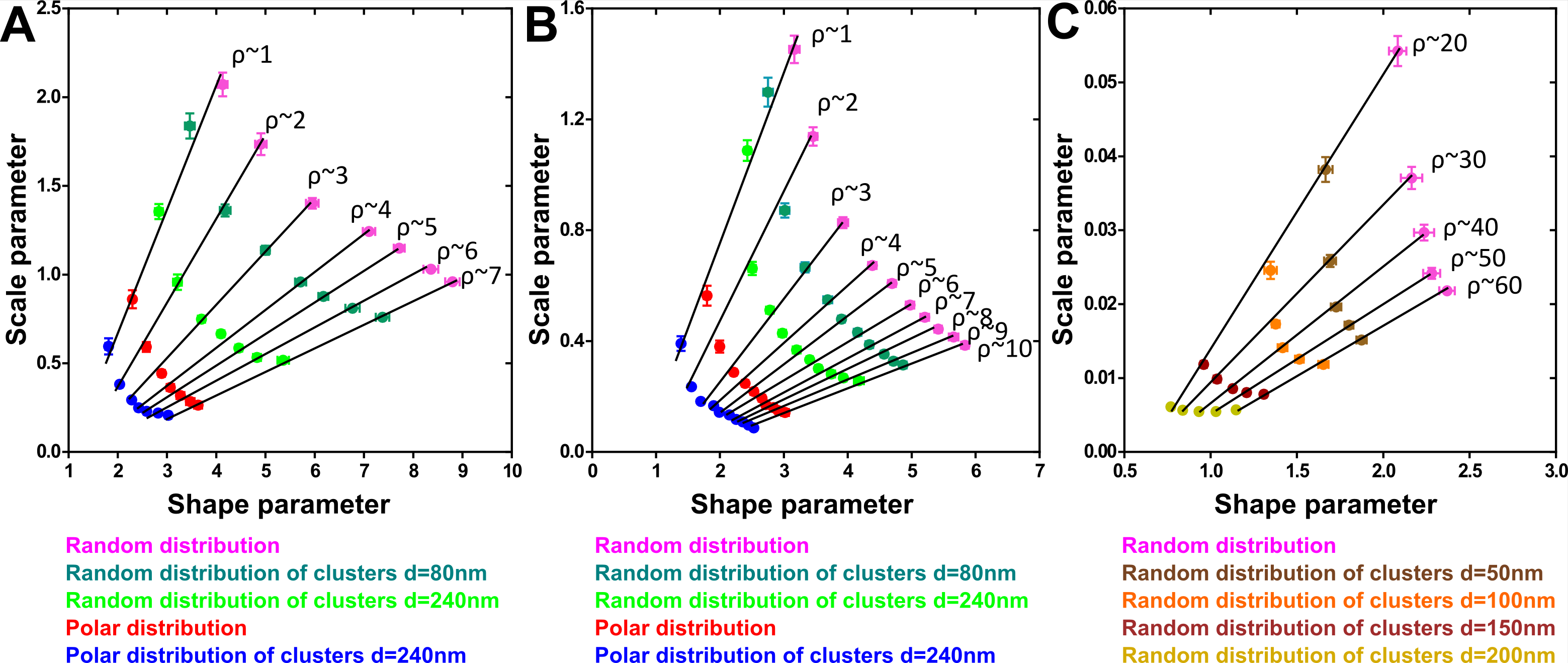

Supplement: S5 Fig — (A) Reference graph for WF and TIRF images. (B) Reference graph for SIM images. (C) Reference graph for PALM images. The densities, ρ (tiles/μm2), of the simulated microscopy images are indicated in the graph. (TIF) [file pcbi.1005095.s005.tif]

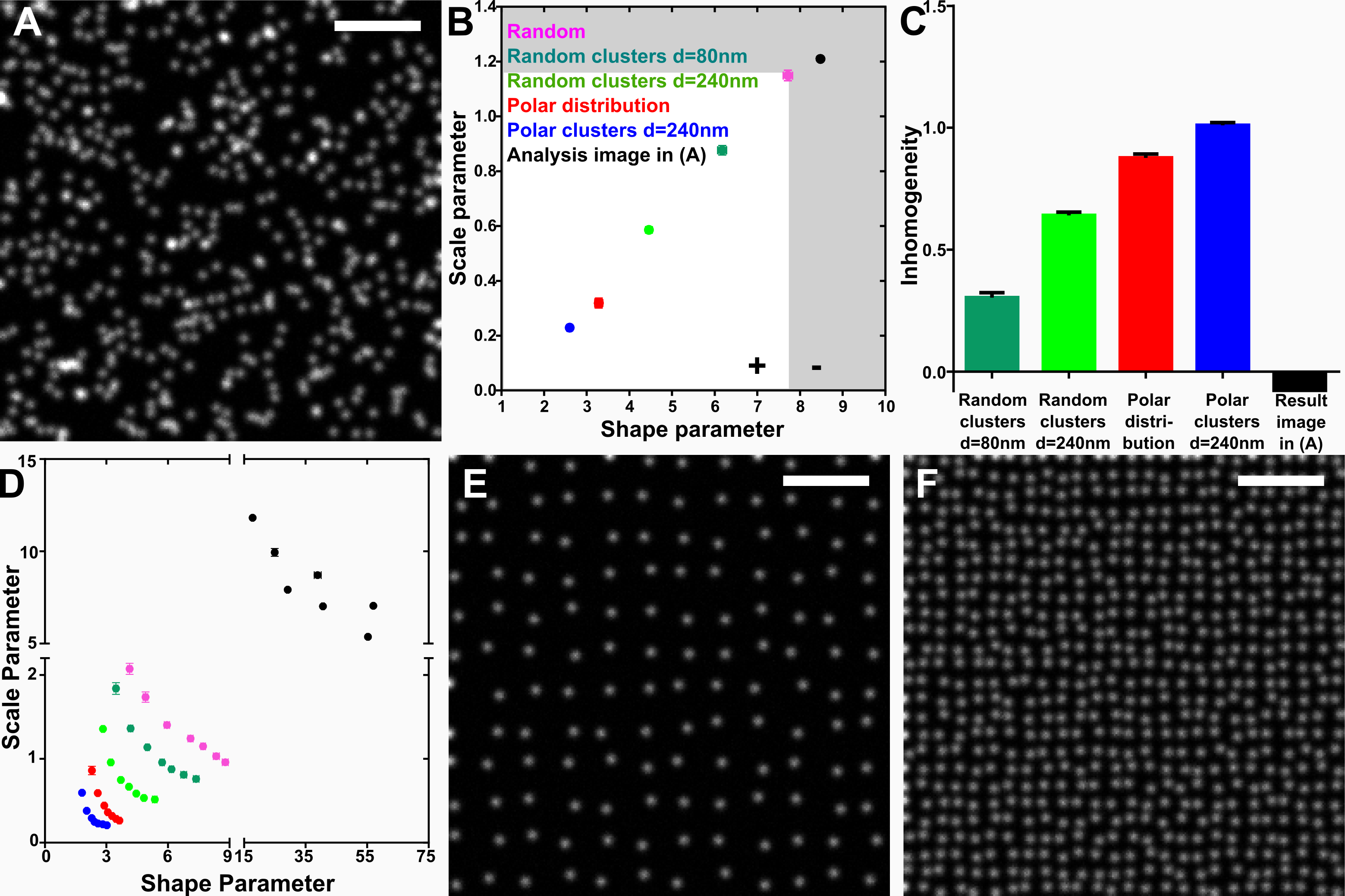

Supplement: S6 Fig — (A) Simulated WF image of a random pattern with density ρ = 5 (tiles/μm2). (B) Shape and scale parameters characteristic for images with density ρ = 5 (tiles/μm2) are plotted for the various point patterns (random, random clusters with diameter d = 80 nm, random clusters with diameter d = 240 nm, polar and polar clusters with diameter d = 240 nm). The white part of the graph represents the area where the deviation from random is calculated as a positive value. Results falling in the grey part of the graph, however, have negative values and underline the fact that the shape and scale parameters are larger than those for the reference of a random distribution. In this graph, the black point is the result from analysis on the image in (A). (C) The inhomogeneity measure increases from clustered to polar patterns. The black bar has a negative inhomogeneity value because the result from analysis on the image in (A) has shape and scale parameters larger than the random distribution reference point. (D) Plot of shape and scale parameters, including the results from analyzing images with regular patterns of density 1 ≤ ρ ≤ 7 (tiles/μm2) (black points, example images shown in E and F). Each point represents the average (and SEM) of 50 simulated WF images for each patter and density. (E) Simulated WF image of a regular pattern with density ρ = 1 (tiles/μm2). (F) Simulated WF image of a regular pattern with density ρ = 7 (tiles/μm2). Scale bar in (A, E, F): 2 μm. (TIF) [file pcbi.1005095.s006.tif]

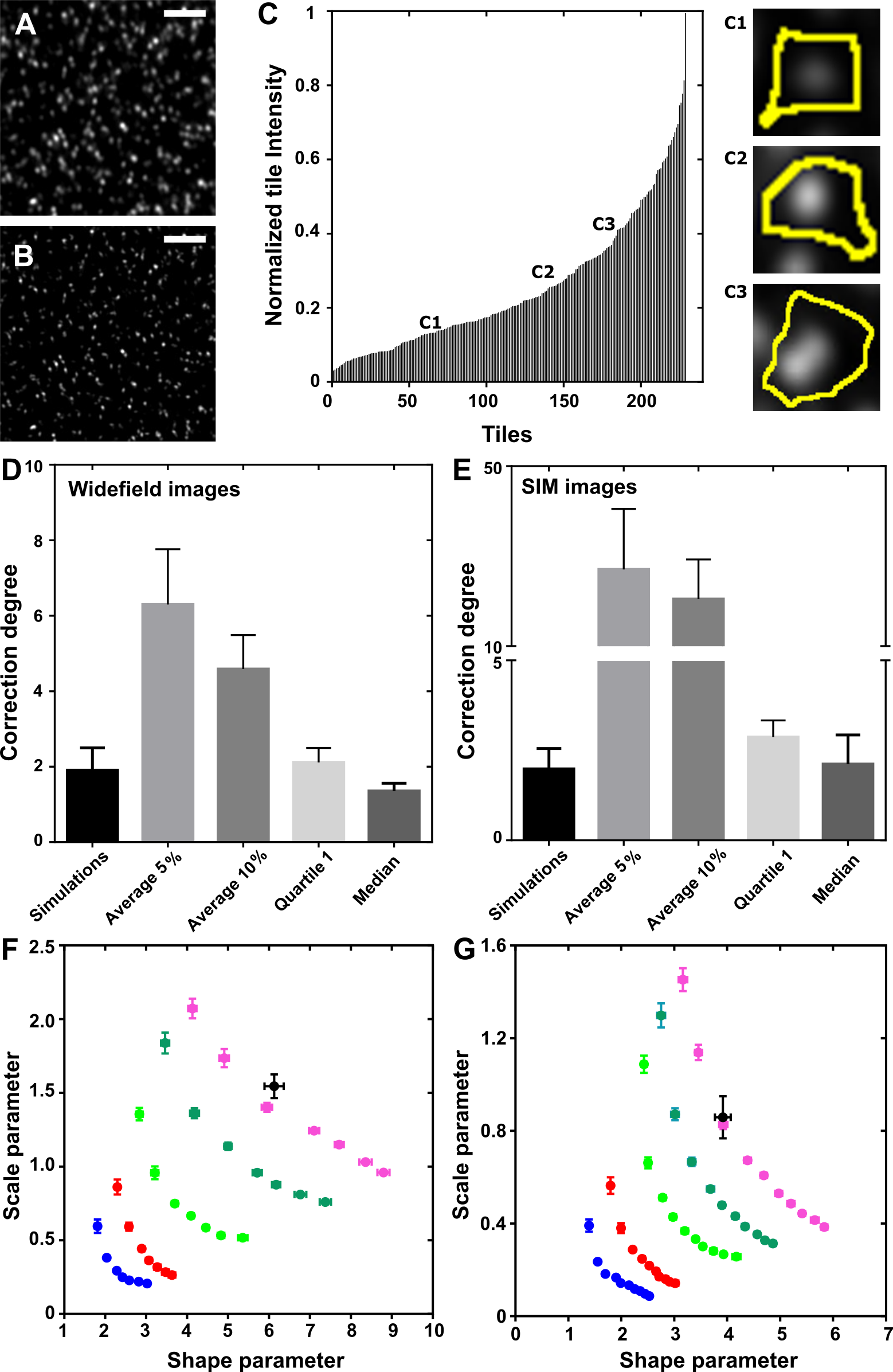

Supplement: S7 Fig — To imitate biological samples, test samples of cell surface receptor staining were prepared. (A) WF and (B) SIM images of randomly dispersed EGFR primary antibody stained with goat anti-mouse Alexa-488 on coverslips. Scale bars: 2 μm. (C) Normalized tile intensity distribution of (A). Intensity of tiles C1-3 is indicated in the graph. C1-3 are examples of tiles containing the detected single unit (C1), two and three units (C2 and C3, respectively) taken from the image in (A). (D, E) Correction degree. Images of WF (D) and SIM (E) were analyzed with QuASIMoDOH by using a correction factor of the 5% average of the lowest tile intensity values (Average 5%), the 10% of the lowest tile intensity values (Average 10%), the first quartile (Quartile 1), and the median (Median) of the tile intensity values. Black bar labeled ‘Simulations’ shows the comparison to simulated images (see as well S4 Fig). The average (and SEM) of 25 images is displayed (percentage of pixels above the threshold ranging from 5% to 25%). First quartile and median, for WF and SIM images, respectively, seem to provide the best guess for C from the image, given the same degree of correction as used in the simulations. (F, G) QuASIMoDOH analysis results of WF (F) and SIM (G) images obtained using first quartile and median, respectively, as correction factors. (TIF) [file pcbi.1005095.s007.tif]

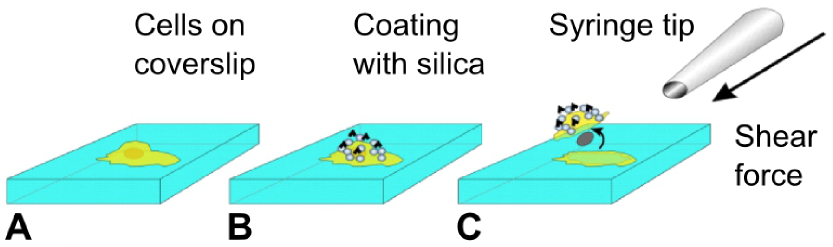

Supplement: S8 Fig — (A) Cells are first seeded and grown on a coverslip. (B) Cells are subsequently incubated with silica beads to coat the apical membrane. (C) After hypotonic swelling, the coated apical membrane is then removed by applying shear force. (TIF) [file pcbi.1005095.s008.tif]

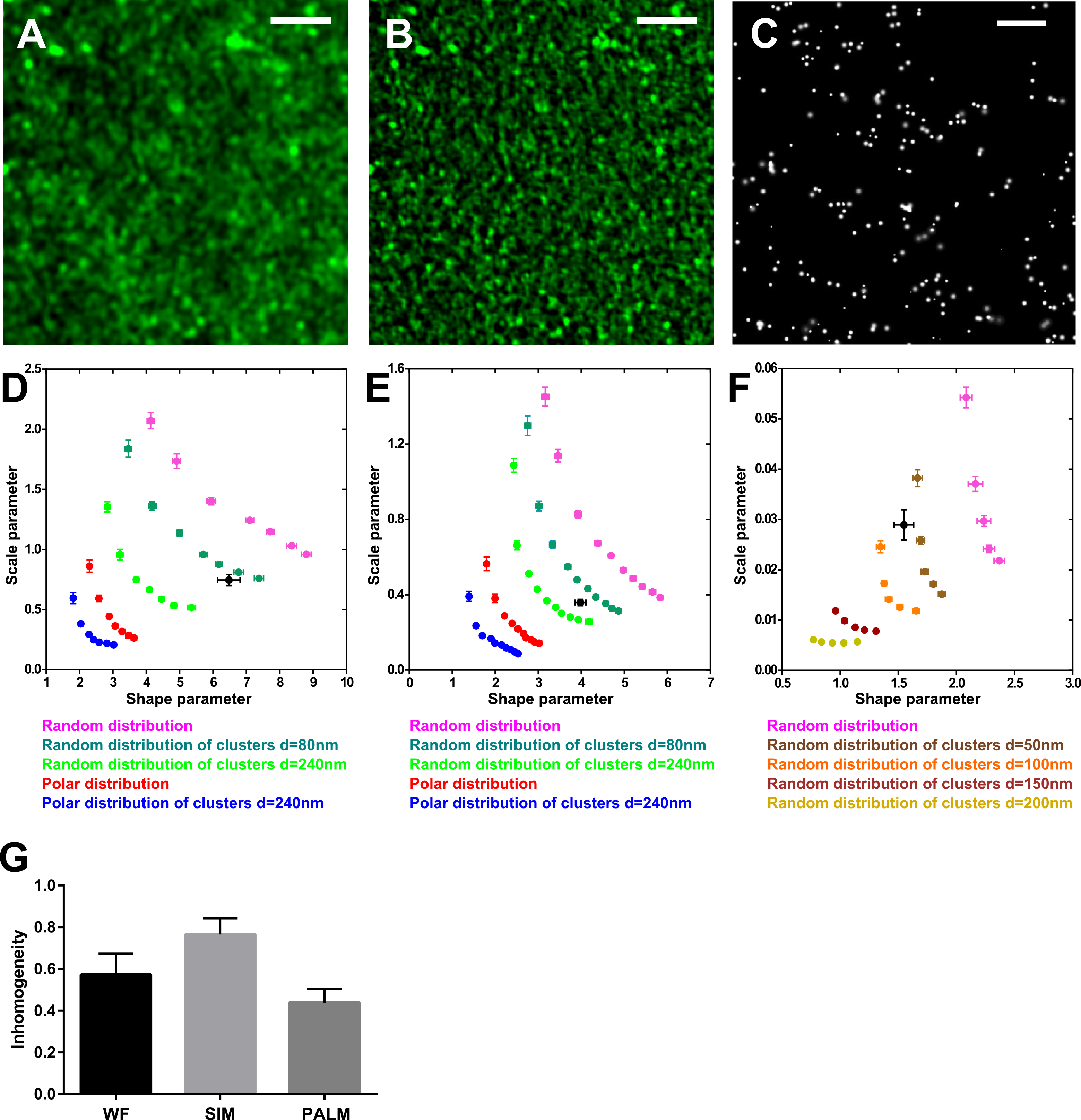

Supplement: S9 Fig — Analysis of the distribution of GPI on the surface of MDA-MB-468 cells. (A) WF and (B) SIM image of GPI-GFP on a supported plasma membrane sheet. (C) Rendered PALM image of paGFP-GPI. Analysis of the GPI distribution from (D) WF, (E) SIM, and (F) PALM images. (G) Comparison of the inhomogeneity measure. Normalization across densities was carried out between the reference point for a random pattern and clusters with diameter d = 240 nm. Scale bar in (A and B): 2 μm. Scale bar in (C): 500 nm (see Materials and Methods for image details). Error bars in (D-G) represent standard error of the mean. Analysis in (A and B) is based on 25 cells, analysis in (C) is based on 16 ROIs. The average r2 in (A) is 0.82, in (B) is 0.64, and in (C) is 0.74. (TIF) [file pcbi.1005095.s009.tif]

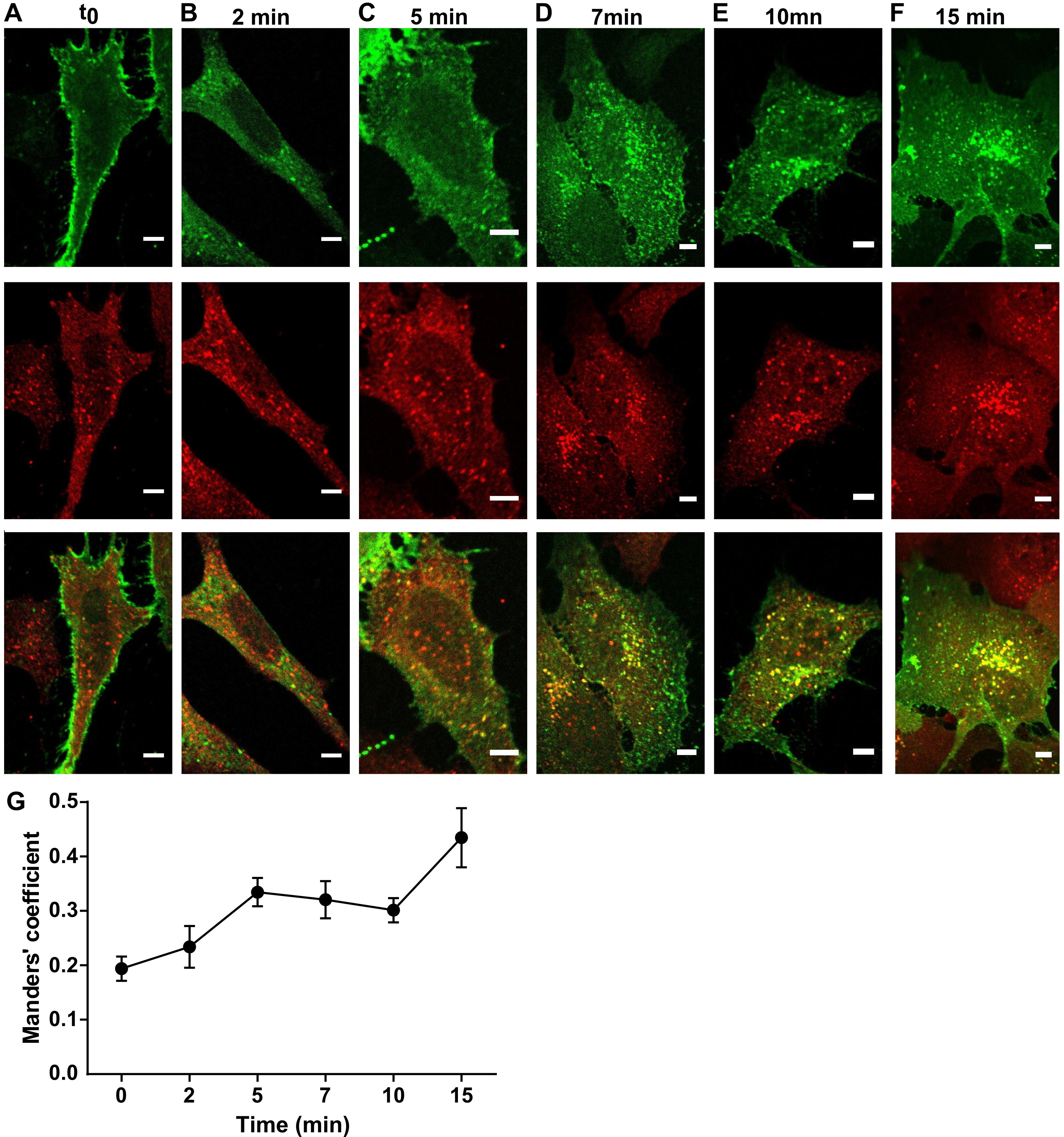

Supplement: S10 Fig — The internalization of EGFR was followed using confocal imaging of HeLa cells (A) before incubation with EGF and (B-F) at 2, 5, 7, 10, and 15 minutes after incubation with 20 ng/ml of EGF. The top panels represent the distribution of EGFR-GFP (in green), the middle panels show the early endosome marker EEA1 (in red), and the bottom panels are images of the merged channels. (G) Quantification of colocalization between EGFR and early endosomes, using Manders’ coefficient. The graph shows an increased colocalization after two minutes of cell incubation with EGF. Scale bars (A-F): 5 μm. (TIF) [file pcbi.1005095.s010.tif]

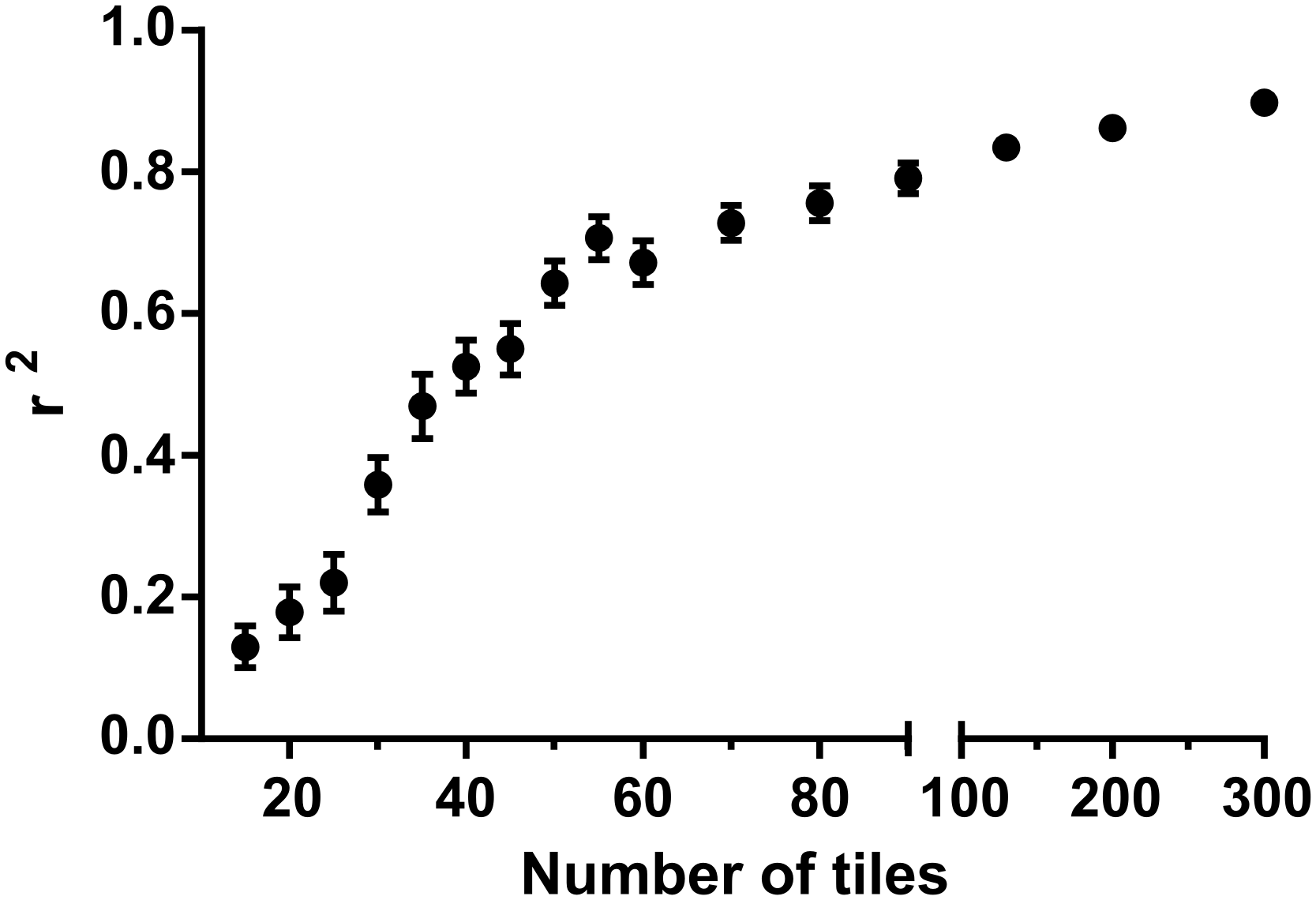

Supplement: S11 Fig — The r2 value increases with an increase in the number of tiles. Analysis based on 60 images per point. Error bars represent SEM. (TIF) [file pcbi.1005095.s011.tif]

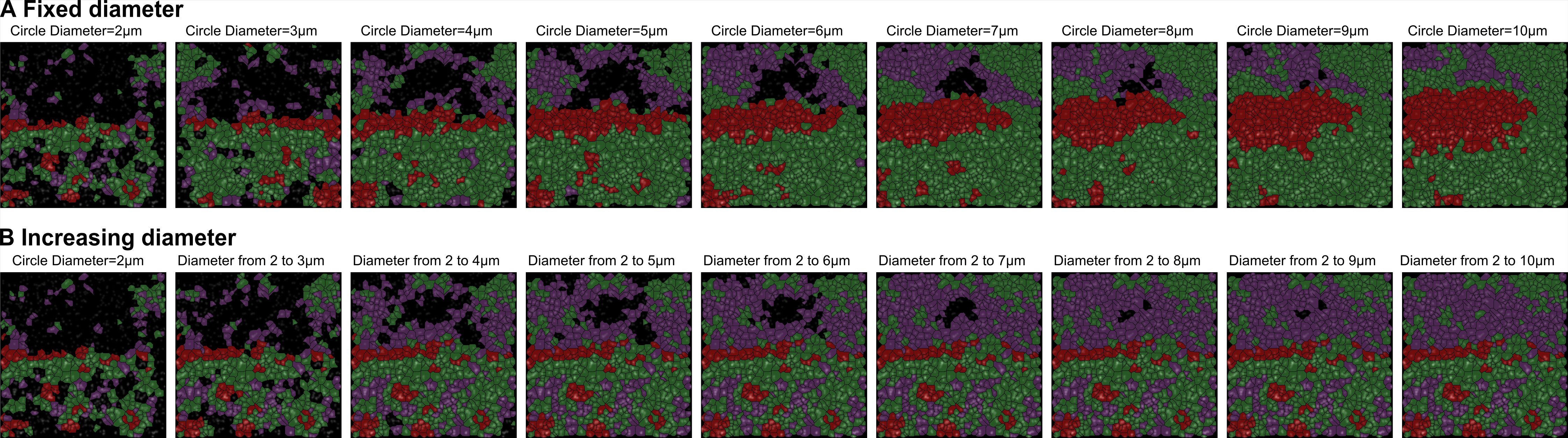

Supplement: S12 Fig — The local analysis results using different diameters (A) and different diameter intervals (B) are displayed for the image shown in Fig 6A. (TIF) [file pcbi.1005095.s012.tif]

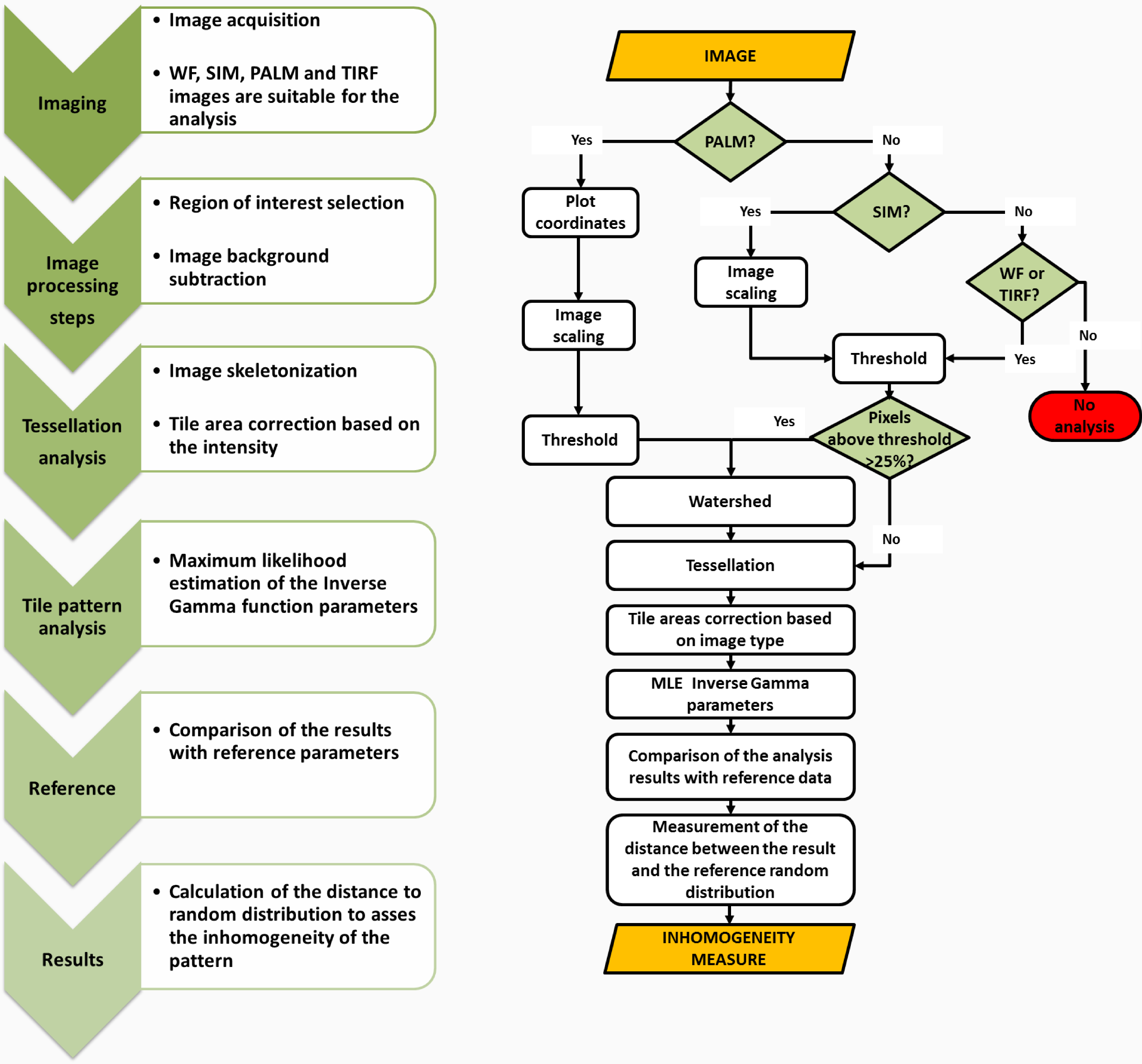

Supplement: S13 Fig — Left graph: analysis steps. Right graph: workflow. (TIF) [file pcbi.1005095.s013.tif]

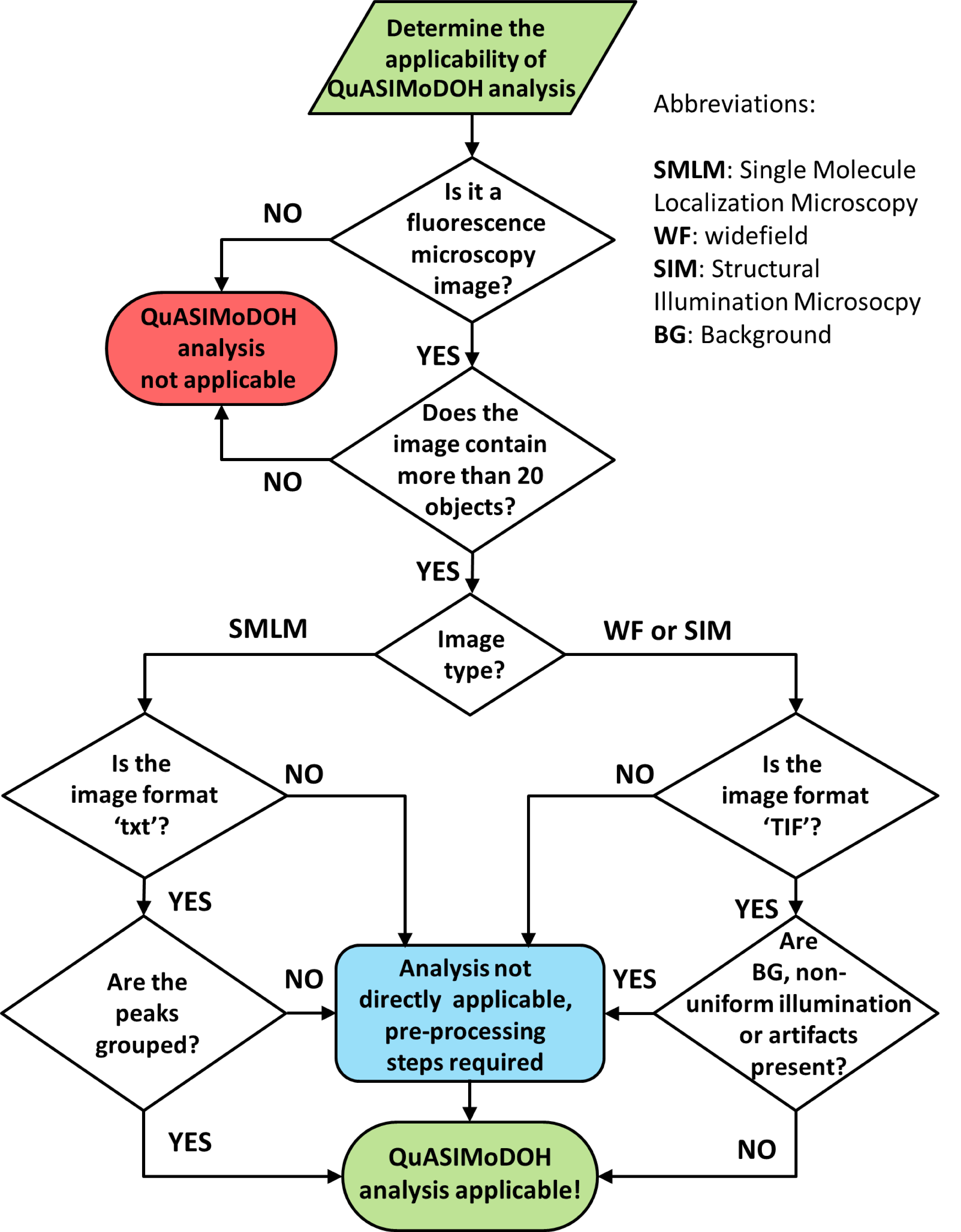

Supplement: S14 Fig — (TIF) [file pcbi.1005095.s014.tif]

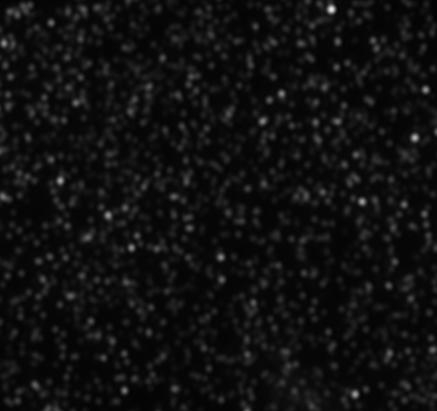

Supplement: S1 File — The folder contains (i) the plugin ‘Quasimodoh_Analysis-1.0.0.jar’ for running QuASIMoDOH using ImageJ/Fiji, (ii) the documentation of the plugin ‘QuASIMoDOH Analysis Documentation.pdf’ and (iii) different datasets in the folder ‘Test Data’. The proteins imaged are indicated. (ZIP) [file pcbi.1005095.s018.zip › S1_File/Plugin and documentation/Test Data/Na+K+ ATPase distribution analysis/Na+K+ ATPase_WF_ROI_BgSubtracted_1.tif]

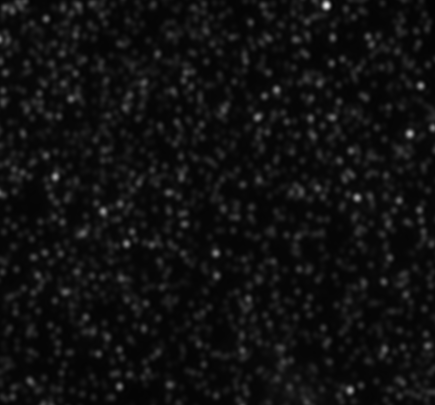

Supplement: S1 File — The folder contains (i) the plugin ‘Quasimodoh_Analysis-1.0.0.jar’ for running QuASIMoDOH using ImageJ/Fiji, (ii) the documentation of the plugin ‘QuASIMoDOH Analysis Documentation.pdf’ and (iii) different datasets in the folder ‘Test Data’. The proteins imaged are indicated. (ZIP) [file pcbi.1005095.s018.zip › S1_File/Plugin and documentation/Test Data/Na+K+ ATPase distribution analysis/Na+K+ ATPase_WF_ROI_BgSubtracted_2.tif]

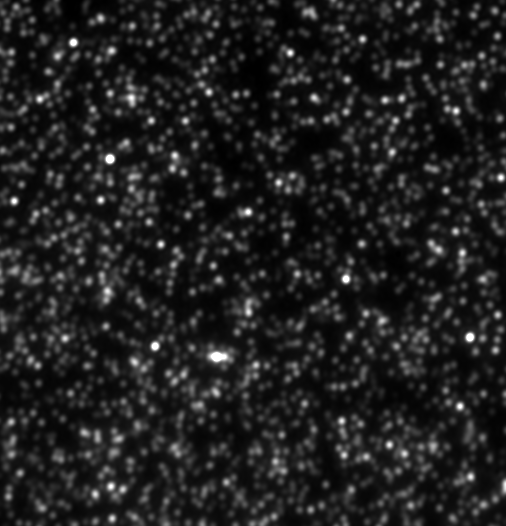

Supplement: S1 File — The folder contains (i) the plugin ‘Quasimodoh_Analysis-1.0.0.jar’ for running QuASIMoDOH using ImageJ/Fiji, (ii) the documentation of the plugin ‘QuASIMoDOH Analysis Documentation.pdf’ and (iii) different datasets in the folder ‘Test Data’. The proteins imaged are indicated. (ZIP) [file pcbi.1005095.s018.zip › S1_File/Plugin and documentation/Test Data/Na+K+ ATPase distribution analysis/Na+K+ ATPase_WF_ROI_BgSubtracted_3.tif]

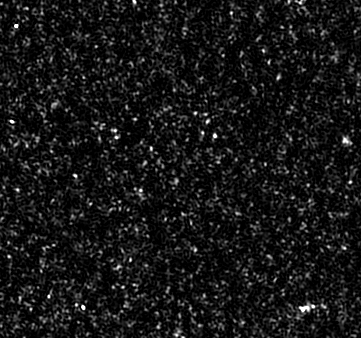

Supplement: S1 File — The folder contains (i) the plugin ‘Quasimodoh_Analysis-1.0.0.jar’ for running QuASIMoDOH using ImageJ/Fiji, (ii) the documentation of the plugin ‘QuASIMoDOH Analysis Documentation.pdf’ and (iii) different datasets in the folder ‘Test Data’. The proteins imaged are indicated. (ZIP) [file pcbi.1005095.s018.zip › S1_File/Plugin and documentation/Test Data/NCT distribution analysis/NCT_SIM_ROI_BgSubtracted.tif]
