# Supplementary material for: Inhomogeneity Based Characterization of Distribution Patterns on the Plasma Membrane
Source: PLoS Comput Biol. 2016 Sep 7;12(9):e1005095. doi: 10.1371/journal.pcbi.1005095 (PMC5014321; doi:10.1371/journal.pcbi.1005095)
Supplement: S1 Table — (PDF) [file pcbi.1005095.s015.pdf]

**PDFs**	**r^2**
Inverse Gamma	0.990555
Log Normal	0.981851
Weibull	0.979905
Lorentzian	0.975521
Extreme Value 4-parameter Tailed	0.958656
Gamma	0.957773
Gaussian-Lorentzian Cross Product	0.947562
Exponential To Max Power Peak	0.944692
Extreme Value 4-parameter Fronted	0.933613
Chi square	0.933272
Extreme Values	0.923774
Gaussian	0.905187
Error	0.902568
Logistic	0.894448
Pearson VII	0.893375
Student	0.893139
Exponentially Modified Gaussian	0.871573
Laplace	0.85151
Asymmetric Logistic Peak	0.842441
Symmetric Double Sigmoidal	0.815405
