# Supplementary material for: Inhomogeneity Based Characterization of Distribution Patterns on the Plasma Membrane
Source: PLoS Comput Biol. 2016 Sep 7;12(9):e1005095. doi: 10.1371/journal.pcbi.1005095 (PMC5014321; doi:10.1371/journal.pcbi.1005095)
Supplement: S1 Text — The supplementary text provides additional information about the creation of discrete point pattern images, simulating the imaging process, threshold selection, determination of the probability density function, determination of the correction factor, and local analysis: circle size determination. (DOCX) [file pcbi.1005095.s016.docx]

**S1 Text: Details of QuASIMoDOH analysis development**

**Inhomogeneity based characterization of distribution patterns on the plasma membrane**

Laura Paparellia,b,c, Nikky Corthouta,c,d, Benjamin Paviea,c, Devin L. Wakefielde, Ragna Sannerudb,c, Tijana Jovanovic-Talismane, Wim Annaertb,c,*, Sebastian Muncka,c,d,*

a VIB Bio Imaging Core, Herestraat 49, Box 602, 3000 Leuven, Belgium.

b Laboratory of Membrane Trafficking; Department of Human Genetics, KU Leuven, Herestraat 49, Box 602, 3000 Leuven, Belgium.

c VIB Center for the Biology of Disease, KU Leuven, Herestraat 49, Box 602, 3000 Leuven, Belgium.

d VIB, LiMoNe, Herestraat 49, Box 602, 3000 Leuven, Belgium.

e Department of Molecular Medicine, Beckman Research Institute of the City of Hope Comprehensive Cancer Center, Duarte, California, USA.

*Correspondence should be addressed to S. M. Sebastian[.munck@cme.vib-kuleuven.be](mailto:munck@cme.vib-kuleuven.be) or W. A. Wim.Annaert@cme.vib-kuleuven.be.

**Creation of discrete point pattern images**

The images of point patterns were established considering a two-dimensional point process $P$ of individual points $p$*,* represented as individual pixels, distributed on a support with homogeneous background:$P=\sum_{i=1}^{N} p_{i}$. We assumed $P$ to be a homogeneous, or a clustered, or an inhomogeneous point process [1]. All images were created in a similar way (S3 Fig).

A random Poisson pattern was generated by using a random number generator over the image matrix. Clusters, resulting from the grouping of points within the process, can arise from a seeding point process (Matérn process [1]), in which each point $p\in P$ is replaced by a random finite set of points *Z_p_* within the cluster associated with *p*. The superposition of all clusters yields the process $Y=P*Z_{p}$*,* with $Z_{p}$ being independent. Thus, clusters are generated by an additive point process, where areas around seed points were set as regions of clusters with a probability to hold (additional) points (for simplicity in the simulations we only considered homogeneous additive point processes). Since the additive point process is a random Poisson process, variability in the cluster size is introduced in each image. Cluster diameters were represented by the diagonal of the square area containing the clustered points. Gradients were generated by increasing the density of random points (this pattern is referred to as a ‘polar distribution’) or clustered points (this pattern is referred to as ‘polar clusters’). In both cases, the density linearly increased in ten steps across the image (S3 Fig). Note that the generated images are not a true simulation of the biology, but simply a means to generate a practical set of test images with different patterns.

**Simulating the imaging process**

Simulations were performed using MATLAB 2012b (Mathworks, MA USA) and the MATLAB toolbox Dip*image* 2.4.1 (TU Delft). Images of point distributions were generated as described above. The blurring of the image was simulated by the convolution with the PSF. Since we analyzed 2D-images, for simplicity, a Gaussian profile with a full-width half maximum (FWHM) similar to the expected FWHM of the PSF was used. The FWHM of the Gaussian profile is equal to $2*\sqrt{2*\ln\left( 2 \right)}\sigma$, with $\sigma$ being the standard deviation, which is approximated by 2.355$\sigma$. Based on the microscopy technique, different resolutions were simulated: 200 nm for widefield microscopy, 100 nm for SIM images, and 20 nm for images acquired by single molecule super-resolution techniques. We assumed fluorescent molecules and staining procedures to be of equal brightness. Next, to simulate photon counting noise, Poisson noise was added to the blurred images using MATLAB functions: “noise,” choosing “Poisson’’ as noise type, and setting the number of photons equal to 4000 for widefield images and 500 for SIM images. Finally, background noise was added in the form of Gaussian noise (S1 Fig).

**Threshold selection**

Thresholding was used to separate signal from background. We performed this step by selecting one of the automatic thresholds implemented in ImageJ/Fiji [2]. The selection of the threshold type was based on its ability to binarize the image and detect the majority of the signal. The selection of the threshold is made by the user based on their judgment, which intensities are signal and which intensities are background. A convenient method for choosing the threshold is reported in the paper *‘Qualitative and Quantitative Evaluation of Two New Histogram Limiting Binarization Algorithms. Jan Brocher. International Journal of Image Processing (IJIP). Volume - 8   Issue - March 2014’* [3]. The techniques described in this paper allow for a fast and easy-to-use evaluation of different automatic intensity segmentation algorithms. The final selection of the most appropriate threshold is, for all cases, semi-automatic since the user must indicate a reference intensity cutoff value. This cutoff value can, however, be assessed in a separate experiment.

**Determination of the probability density function**

The Inverse Gamma PDF was selected based on data reported in the literature and on practical investigations. The generalized Gamma distribution has been used earlier for interpreting tessellation data of homogeneous Poisson distributions [4,5], and recently the Inverse Gamma probability density function was used to model inhomogeneous Poisson distributed data from Voronoi tessellations in the context of earthquake data [6]. We also identified the Inverse Gamma as the best PDF for modeling image tile areas (S1 Table). Specifically, this was the result of plotting histograms of the tile areas (obtained from analyzing different point patterns with different densities) and subsequently applying 20 PDFs to the data (S1 Table). We normalized the histograms with the number of bins set to the square root of the number of tiles. From a calculation of the coefficient of determination (r^2^) for each PDF fit, we identified, in agreement with literature, that the Inverse Gamma PDF was the best function to describe the data.

**Determination of the correction factor**

The correction factor is the intensity of a fluorescent entity in the image, like a single fluorophore or a group of fluorophores, used to correct tile areas containing multiple unresolvable entities. As such, the correction factor could be determined in a separate experiment. However, the stochasticity in the image results in a mixture of both dense and more isolated points allowing to estimate a fluorescent detectable unit, dependent on the resolution and sensitivity of the microscope/camera, that can be used as a correction factor. Since this stochasticity is also given for clusters consisting of random units and polar distributions, we were able to estimate an image based correction factor from the set of tile intensities.

First, we tested the influence of the resolution on the correction degree (ratio between the number of areas obtained by tile size correction and the number of tiles). We used simulated WF and SIM images of random distribution with different resolution (S4A-E Fig), where the intensity of a single point was used for the correction. With the change in resolution on the scale tested being much smaller than the average distance between the points, no significant change in correction degree was detected.

Next, we tested if the correction factor can be estimated from the tile intensity distribution (S4F Fig) without the knowledge of the intensity of a single point in simulated images. We determined the best estimate by measuring the accuracy to provide the correct number of known points. The accuracy is defined as the ratio between the number of tile areas, obtained by tile size correction, and the total number of points in the image. Four different parameters were tested for the estimation of the correction factor: the minimum tile intensity, the 5% average of the lowest intensity values, the 10% average of the lowest intensity values, and the first quartile. We also calculated the accuracy of the analysis performed without applying a tile area correction. Based on the resulting accuracy, we selected the correction factor of the 5% average of the lowest intensity values for simulated widefield images (S4G Fig), and the 10% average of the lowest values for simulated SIM images (S4H Fig). Remarkably, when no correction is performed for simulated widefield images, the actual number of points in the images is underestimated by ~70%. For PALM image analysis, we simulated the rendered images. Since the pixel based thresholding might still detect objects comprised of neighboring pixels, a moving average was thus used to overcome potential effects of small bordering tiles.

We note that real images are more complex than our simulations. For example, differences in the dynamic range of the detector and variable fluorophore labeling of secondary antibodies are among the possibilities for this complexity. Thus, we expected that intensity steps of the tile intensities for increasing numbers of fluorophores would be less pronounced. To create random test samples that emulate biological cell surface receptor staining, we precipitated epidermal growth factor receptor (EGFR) primary antibodies on coverslips and stained them according to our standard immunohistochemistry protocol. S7 Fig shows the resulting WF (S7A Fig) and SIM (S7B Fig) images, as well as the tile intensity distribution (S7C Fig). The correction factor for analyzing fluorescence microscopy images was selected based on similarity of the correction degree for simulated images (S7D, E Fig). Testing the correction factor estimates as in the simulated images, the lower quartile and the median of the intensity distribution were found to serve as the most suitable correction factors for WF/TIRF and SIM, respectively (S7D, E Fig). Applying the selected correction factors, QuASIMoDOH analysis accurately identified the randomly dispersed antibodies in WF and SIM images as random (S7F, G Fig).

For PALM image analysis, we plotted the coordinates of the grouped reconstructed peaks (see Materials and Methods, ‘Data processing’). Consecutive tiles were corrected using the same intensity per peak to correct tiles containing adjacent peaks.

**Local analysis: circle size determination**

Different circle sizes were systematically tested to optimize the detection of correct local spatial distributions in images (Fig 6A-C). Results were quantified by plotting the percentage of the tiles in the upper third and lower third of the image in Fig 6A correctly assigned as random and clustered distributions, respectively (Fig 6C). The analysis revealed that the detection of a random pattern in the upper part of the simulated images is maximized by using a circle 4 µm in diameter. For the detection of clusters in the lower part of the images, more than 50% of the correctly assigned tiles resulted from using any radius between 2 to 15 µm. To maximize the number of tiles with detected distributions, the analysis was iterated with circles of diameters ranging from 4 to 10 µm (about half of the image) (Fig 6D) and a step size of 1 µm. The procedure of fixing a minimum diameter and stepping up to larger diameters allowed for a higher coverage of assigned tiles than if a single fixed diameter was used, as unassigned tiles are supplemented with distributions detected from larger diameters by this approach (see S12 Fig).

**Supplementary text bibliography**

1. Baddeley A. Spatial Point Processes and their Applications. Stochastic Geometry. Springer Berlin Heidelberg; 2007. pp. 1–75.

2. Schindelin J, Arganda-Carreras I, Frise E, Kaynig V, Longair M, Pietzsch T, et al. Fiji: an open-source platform for biological-image analysis. Nat Methods. 2012;9: 676–682. doi:10.1038/nmeth.2019

3. Jan Brocher. Qualitative and Quantitative Evaluation of Two New Histogram Limiting Binarization Algorithms. Int J Image Process IJIP. 2014;8: 30–48.

4. Hinde AL, Miles RE. Monte carlo estimates of the distributions of the random polygons of the voronoi tessellation with respect to a poisson process. J Stat Comput Simul. 1980;10: 205–223. doi:10.1080/00949658008810370

5. Tanemura M. Statistical distributions of Poisson voronoi cells in two and three dimensions. Forma. 2003;18: 221–247.

6. Barr CD, Schoenberg FP. On the Voronoi estimator for the intensity of an inhomogeneous planar Poisson process. Biometrika. 2010;97: 977–984. doi:10.1093/biomet/asq047
