# Supplementary material for: Inhomogeneity Based Characterization of Distribution Patterns on the Plasma Membrane
Source: PLoS Comput Biol. 2016 Sep 7;12(9):e1005095. doi: 10.1371/journal.pcbi.1005095 (PMC5014321; doi:10.1371/journal.pcbi.1005095)
Supplement: S1 File — The folder contains (i) the plugin ‘Quasimodoh_Analysis-1.0.0.jar’ for running QuASIMoDOH using ImageJ/Fiji, (ii) the documentation of the plugin ‘QuASIMoDOH Analysis Documentation.pdf’ and (iii) different datasets in the folder ‘Test Data’. The proteins imaged are indicated. (ZIP) [file pcbi.1005095.s018.zip › S1_File/Plugin and documentation/QuASIMoDOH Analysis Documentation.pdf]

QuASIMoDOH ANALYSIS DOCUMENTATION

Here we provide a short guide for using QuASIMoDOH: **Qu**antitative **A**nalysis of the **S**patial distributions in **I**mages using **Mo**saic segmentation and **D**ual parameter **O**ptimization in **H**istograms. This guide refers to the Fiji plugin, provided with test data, to help with implementation of QuASIMoDOH analysis.

CONTENTS

Background 1

Image processing steps to apply before running QuASIMoDOH analysis 2

- **A. Processing of widefield, SIM, and TIRF images** 3
- **B. Preparation of PALM data** 4

Description of provided test data 5

How to run QuASIMoDOH analysis 6

- **A. Setting up the work environment** 6
- **B. Global distribution analysis** 7
  - **B1. Global distribution analysis of widefield, SIM, and TIRF images** 7
  - **B2. Global distribution analysis of PALM data** 10
- **C. Local distribution analysis** 12

Appendix 15

Background

QuASIMoDOH is a tool for the quantitative analysis of protein and lipid distributions on the cell surface. QuASIMoDOH can help define patterns in the organization of specific plasma membrane components, and can thus be used to measure changes in their distributions. QuASIMoDOH offers rapid discrimination among patterns of random, polar, random clustered, and polar clustered plasma membrane organization.

The analysis can be applied to images acquired by the following techniques:

- Widefield microscopy
- Structured Illumination Microscopy (SIM)
- Total Internal Reflection (TIRF) microscopy, with widefield resolution
- Photoactivatable Localization Microscopy (PALM)

QuASIMoDOH analysis uses the following steps:

- Detection of fluorescence signal by thresholding;
- Tessellation of the image by skeletonization in polygons (tiles);
- Tile area correction for intensity ;
- Analysis of tile area distributions by modeling with the Inverse Gamma probability density function;
- Maximum Likelihood Estimation (MLE) of the Inverse Gamma function parameters: shape and scale;
- Comparison of the obtained parameters with reference data (*a priori* calculated). For images acquired by widefield, SIM, and TIRF microscopy, the reference data are: random distribution, random distribution of clusters with diameter d=80 nm, random distribution of clusters with diameter d=240 nm, polar distribution and polar distribution of clusters with diameter d=240 nm. For PALM data, the reference distributions are: random and clusters with a diameter equal to 50, 100, 150, and 200 nm.

The Inverse Gamma function parameters and their distance relative to the random distribution reference point provide the measure of spatial inhomogeneity for the fluorescence pattern in an image. QuASIMoDOH reports the closest reference distribution to the analyzed image result. The analysis can be applied in a global or local manner (PALM images can only be analyzed by global analysis). In global analysis mode, the MLE of the Inverse Gamma function parameters is performed by analyzing the area of all tiles present in the image. In local analysis mode, the MLE is carried out on tile subsets automatically selected with a circle of user-defined size, centered on each tile in the image.

QuASIMoDOH applicability diagram:

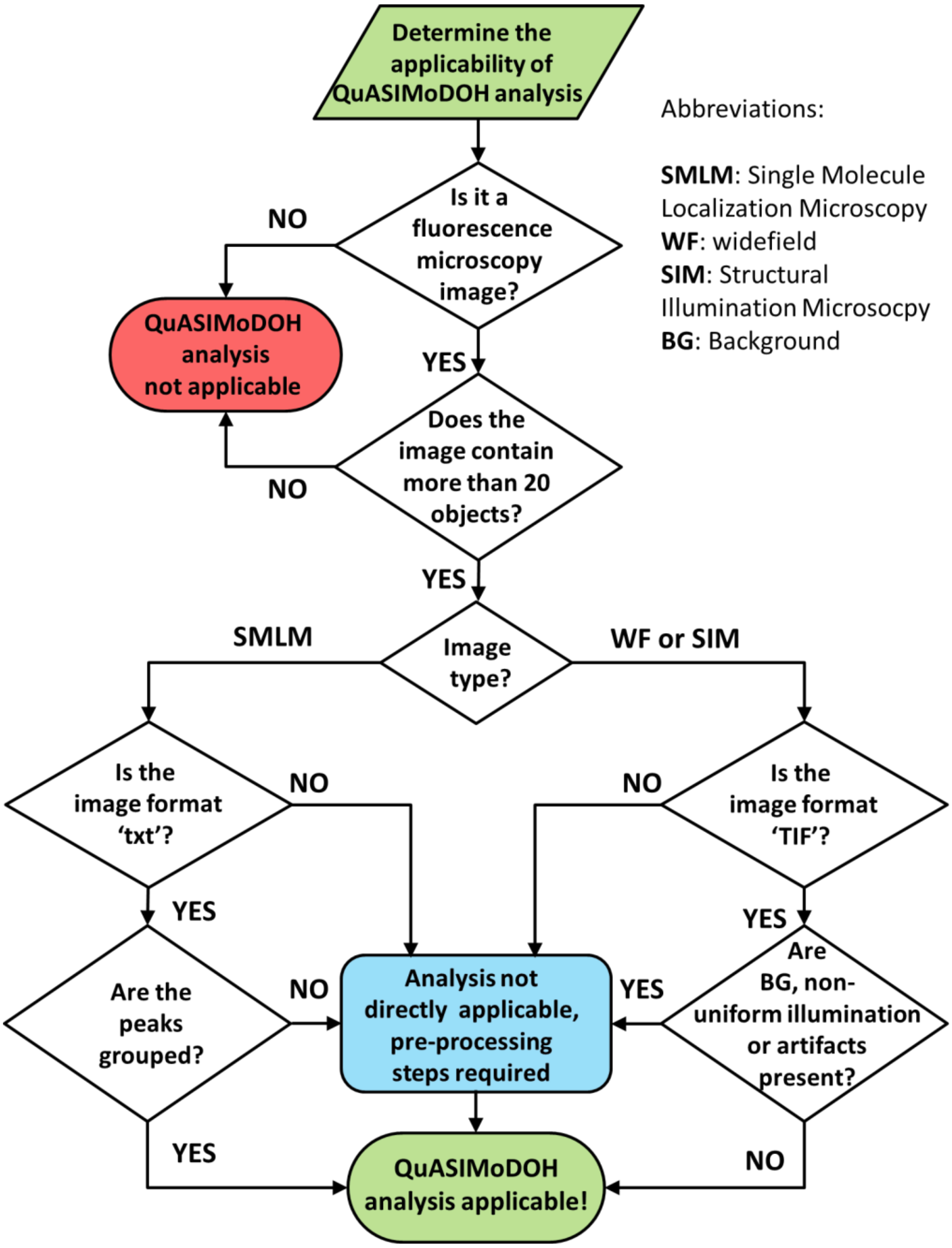

Image processing steps to apply before running QuASIMoDOH analysis

Widefield, SIM, or TIRF image analysis requires a *.tif* image and a few pre-processing steps. PALM image analysis, however, requires a *.txt* file with localized single molecule coordinates from a previously selected region of interest.

A. Pre-processing of widefield, SIM, and TIRF images

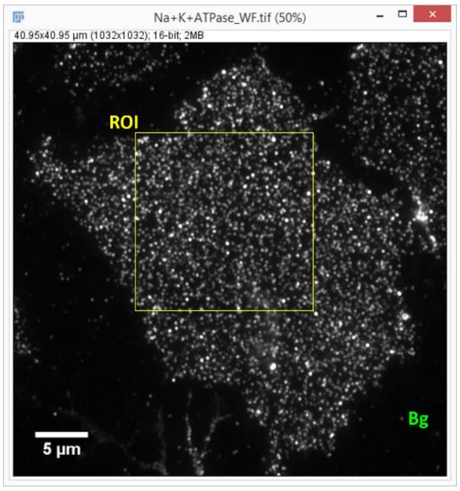

I. Select and crop a region of the plasma membrane to analyze (region of interest, ROI). An image of the sodium-potassium pump (Na^+/K^+ ATPase) is used here.

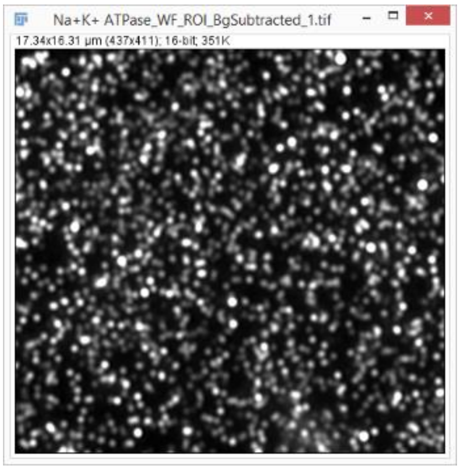

II. Use the following steps to address image background, non-uniform illumination, or artifacts. Background noise can be subtracted, as in the example, by measuring the average intensity of the background from the full image and subtracting this from the average intensity of the ROI (the ROI obtained after background subtraction is shown on the right, middle image). Non-uniform illumination can be removed by flat-field correction. Detailed information on background and non-uniform illumination correction by ImageJ/Fiji can be found in the Fiji guide for Image Intensity Processing: http://imagej.net/Image_Intensity_Processing. The presence of artifacts in the image requires optimization of sample preparation or image acquisition conditions.

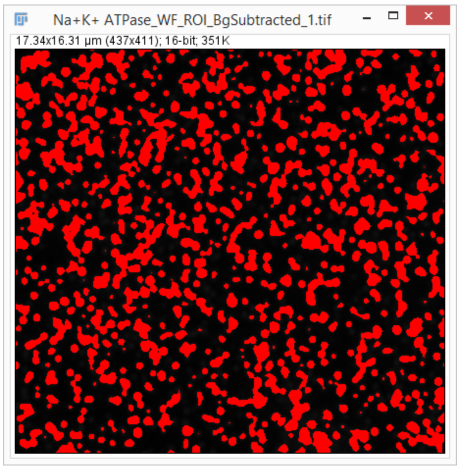

III. Identify an appropriate intensity threshold for separating signal from the background (lower image shown on the right). We use predefined thresholds based on an intensity histogram from the images. In the example, the threshold Li (Li and Tam, 1998) is applied.

B. Preparation of PALM data

PALM localized point coordinates should be saved in a *.txt* file (see left figure below), with the X positions in the first column and Y positions in the second column. The localization precision (Sigma) for each coordinate is shown in the third column. (Note, localization precision is not taken into account for QuASIMoDOH analysis and does not need to be saved with coordinate data). All values can be floating numbers, but they must be tab-separated. Coordinates should be in pixels as QuASIMoDOH analysis will later prompt the user for pixel size (nm/pixel). A binary image of the data is automatically prepared after running QuASIMoDOH analysis (see figure below).

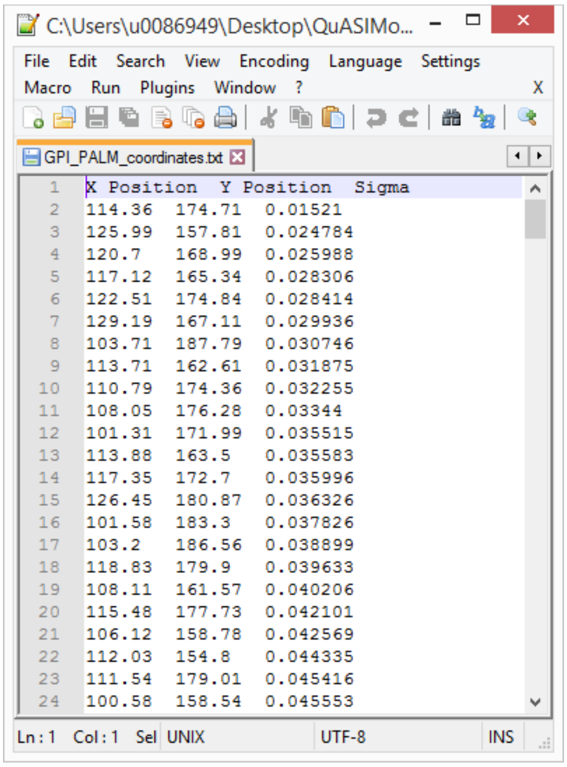

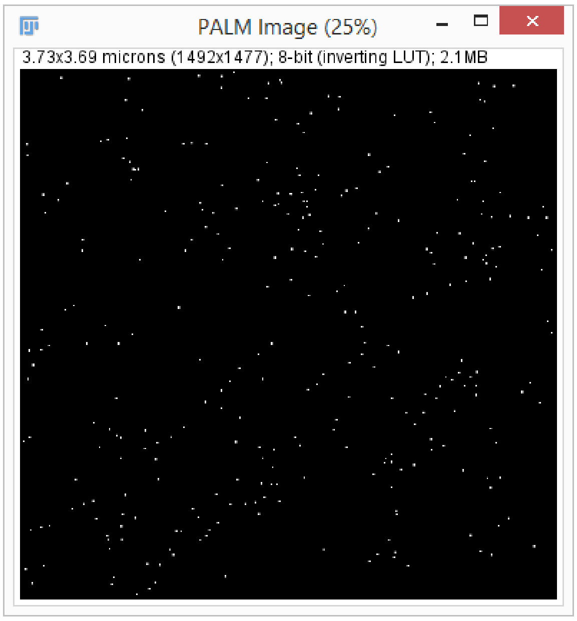

Description of provided test data

We provide the user with data for testing QuASIMoDOH analysis. These include images acquired by widefield and SIM microscopy and coordinates of localized single molecules obtained by PALM.

Regarding widefield and SIM images, we provide ROIs from the original image of the cell surface. Background noise has already been measured and subtracted from these ROI images. Thus, the analysis can be run following the steps provided in the tutorial 'How to run QuASIMoDOH analysis'.

For PALM data, we provide a *.txt* file containing the coordinates of single molecules obtained after peak grouping. As described in the main text, peaks initially localized with Peak Selector software (Research Systems, Inc.) were grouped using a 3 x maximum localization precision and a maximum dark time of 5 s.

Below is a list of the provided data with a short description and the thresholding used for analysis.

NAME	FOLDER TITLE	DESCRIPTION	DATA TYPE	THRES-HOLD
Na+K+ ATPase_WF_ROI_BgSubtracted_1<br>*Na+K+ ATPase_WF_ROI_BgSubtracted_2*<br>*Na+K+ ATPase_WF_ROI_BgSubtracted_3*	Na^+/K^+ ATPase distribution analysis	Widefield images of Na^+/K^+ ATPase on the surface of MEF cells (Immuno-histochemical staining).	Widefield images	Li
NCT_SIM_ROI_BgSubtracted	NCT distribution analysis	SIM image of Nicastrin (NCT)-YFP on the surface of MEF cells.	SIM image	Li
GPI_PALM_coordinates[1]	GPI distribution analysis	PALM localization coordinates of paGFP-GPI transiently expressed in MDA-MB-468 cells. Peaks grouping is described in the text.	PALM	Set as Li by default

1. *Peak coordinates kindly provided by: Tijana Jovanovic-Talisman, Department of Molecular Medicine, Beckman Research Institute of the City of Hope Comprehensive Cancer Center, Duarte, California, USA.*

How to run QuASIMoDOH analysis

QuASIMoDOH analysis has been tested with Fiji Life-Line version, 2013 July 15 for Windows 32bit (version 1.47v). QuASIMoDOH analysis can be performed using standard computing power (e.g., Dell Optiplex7010 with an Intel(R) Core(TM) i7-3770 CPU @ 3.40GHz processor and 4.00 GB RAM, running Windows 7 Professional).

A. Setting up the work environment

To create the complete work environment for QuASIMoDOH analysis, the following is required:

I. Download Fiji Life-Line version, 2013 July 15 (http://fiji.sc/Downloads), 32bit or 64bit depending on your computer.

II. Download the file *ssj-2.5.jar,* for Stochastic Simulation in Java, from http://central.maven.org/maven2/ca/umontreal/iro/ssj/2.5/ssj-2.5.jar.

III. Save the file *ssj-2.5.jar* in the Fiji plugins folder.

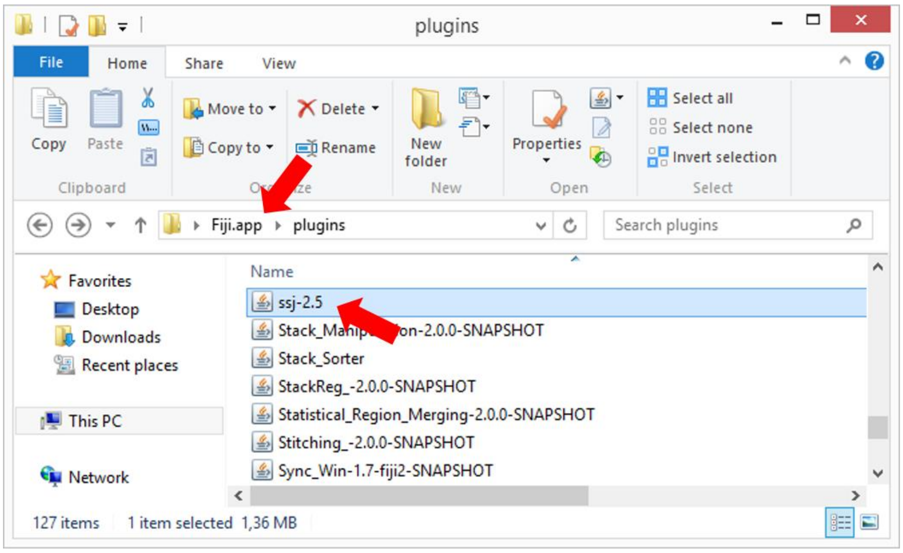

IV. Download the plugin *Quasimodoh_Analysis-1.0.0.jar* and save it to the Fiji plugins folder.

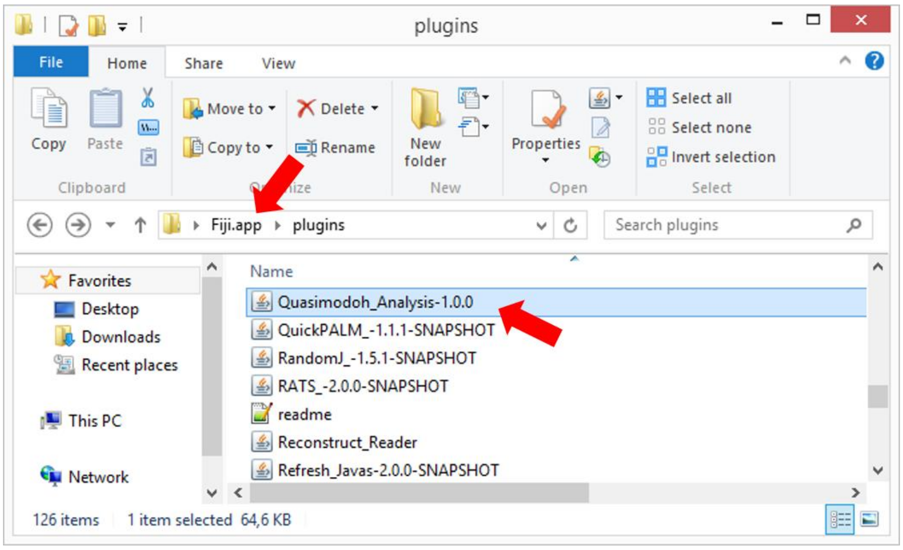

V. Start Fiji.

VI. Install the *Quasimodoh_Analysis-1.0.0.jar* plugin by clicking on Plugins->Install. In the window that appears, select the script and press 'Open'. (Further information regarding the installation of Fiji plugins can be found at the webpage: http://fiji.sc/Installing_3rd_party_plugins).

VII. Restart Fiji. The plugin 'QuASIMoDOH' will be in Plugins->Analyze.

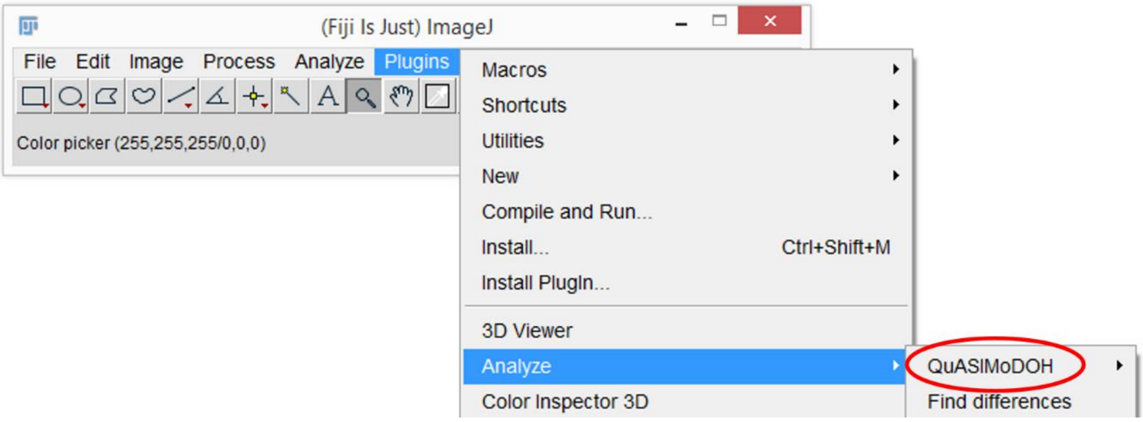

B. Global distribution analysis

To run the global analysis, select 'Global Analysis' from the drop-down menu open from 'QuASIMoDOH'.

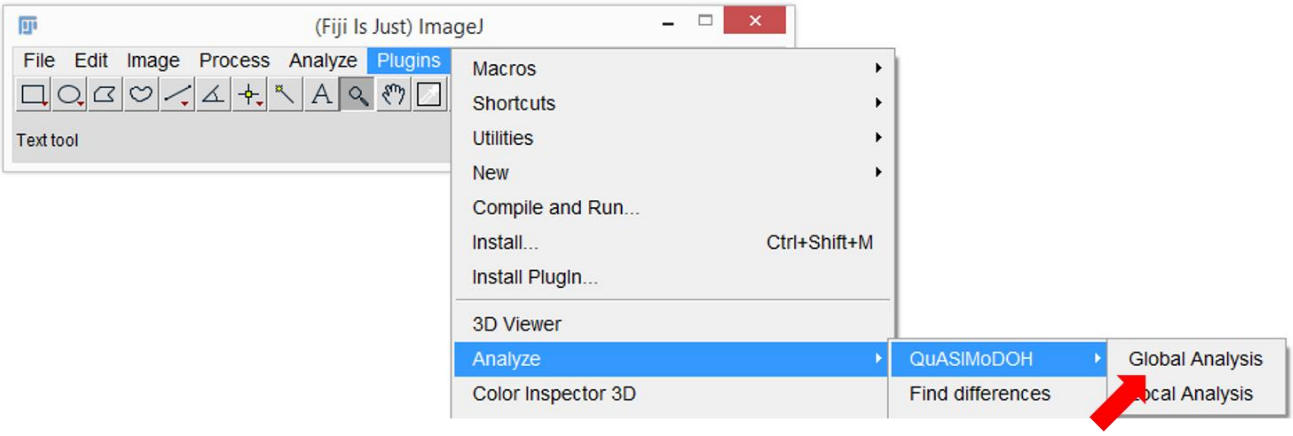

B1. Global distribution analysis of widefield, SIM, and TIRF images

I. Once 'Global analysis' has been selected, a dialog window will appear asking the user to select the folder in which the images are stored.

1. Click on the folder with the images to analyze (in this example 'Na+K+ ATPase distribution analysis' is highlighted, which contains three widefield images).
2. Click on 'Select'.

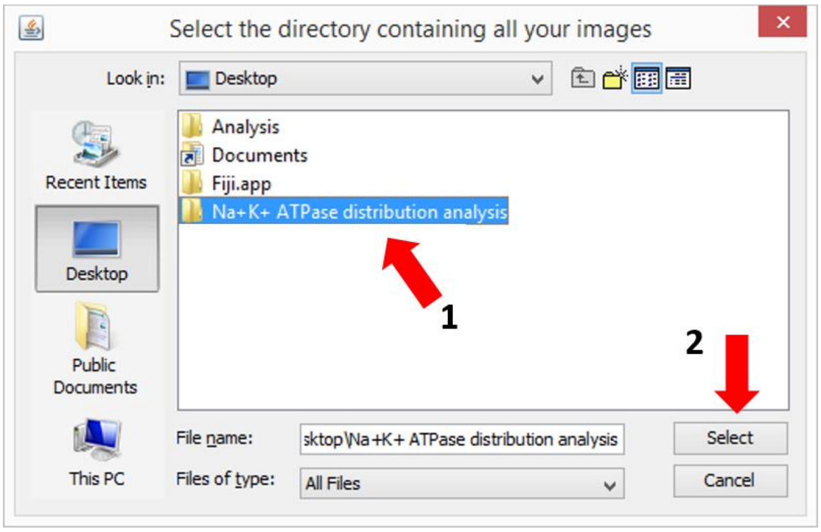

II. A ‘Parameters’ dialog window will appear. Here, parameters for running the analysis can be set as follows:

1. In the window ‘File extension’, enter ‘tif’.
2. Open the drop-down list ‘Image Type’ and select the type of image to analyze based on the microscopy technique used for acquisition. ‘Widefield’ in the example.
3. Open the drop-down list ‘Threshold Type’ and indicate the threshold type previously selected (see above ‘Image processing steps to apply before running QuASIMoDOH analysis’, step III).
4. The graph of the reference distributions with the results will be plotted and saved (as *.png* and *.svg* files). Uncheck the boxes ‘Plot Result Graph’ and ‘Save Result Graph’ if these actions are not desired.
5. Click ‘OK’ to start the analysis.

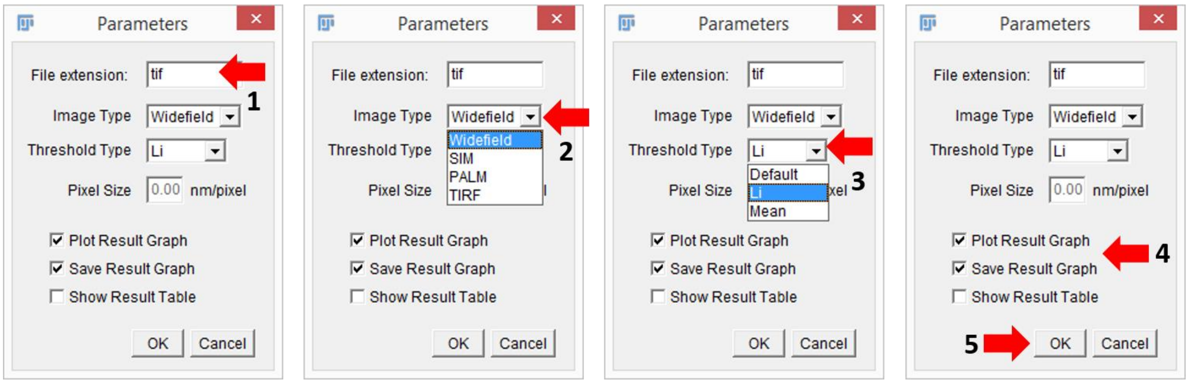

III. The results include (i) the distance of the shape and scale parameters from the reference random distribution, ‘Distance Mean to Random’ (this is the measure of distribution inhomogeneity), and (ii) the closest reference distribution for the result, ‘Closest Distribution’. In the sodium-potassium pump example image, the shape and scale parameter distance to the random distribution is 0.042, and random distribution also happens to be the closest spatial distribution for the pattern of fluorescence in this ROI.

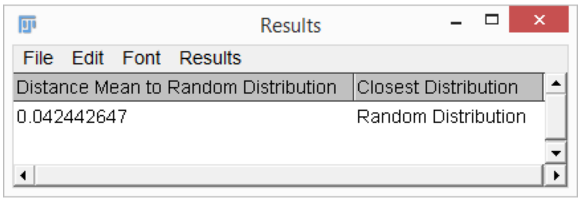

IV. The QuASIMoDOH Analysis Result plot shows the shape and scale parameters of the reference distributions together with the analysis result. The displayed reference data were obtained from analyzing simulated images with the same density of areas obtained after tile size correction (density equals the number of tiles divided by the area of the image in µm^2). The average density of the analyzed images is provided in the plot title. The reference data in the plot belong to the same density range.

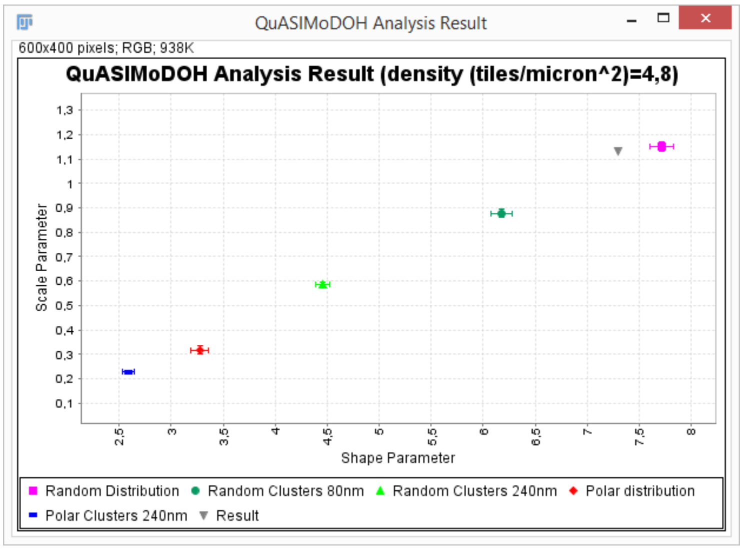

V. By checking the box 'Show Results Table',

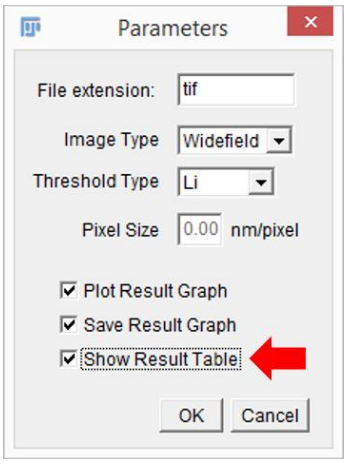

a 'Log' file appears:

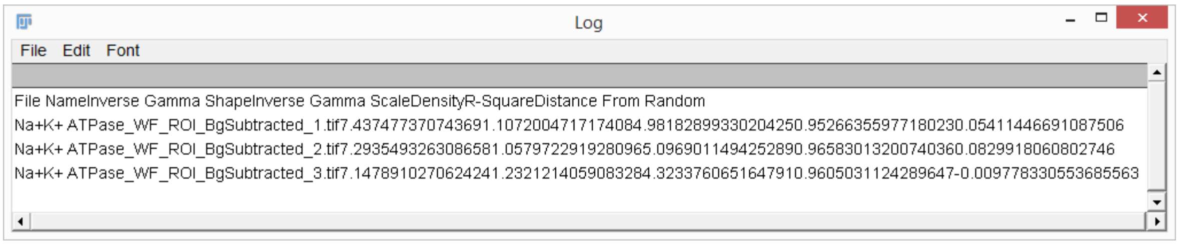

This file contains the following information:

- 'File Name': image title.
- 'Inverse Gamma Shape': Inverse Gamma shape parameter.

– 'Inverse Gamma Scale': Inverse Gamma scale parameter.
– 'Density': density of the corrected tile areas.
– 'R-Square': coefficient of determination obtained from the fitting of the tile areas histogram using the shape and scale parameters.

VI. 'Results' and 'Log' can then each be saved as text (.txt) files from File->Save As

B2. Global distribution analysis of PALM data

I. Once 'Global analysis' has been selected, a dialog window will appear asking the user to select the folder in which the images are stored.

1. Click on the folder with the images to analyze (in this example 'GPI distribution analysis' is highlighted, which contains three widefield images).
2. Click on 'Select'.

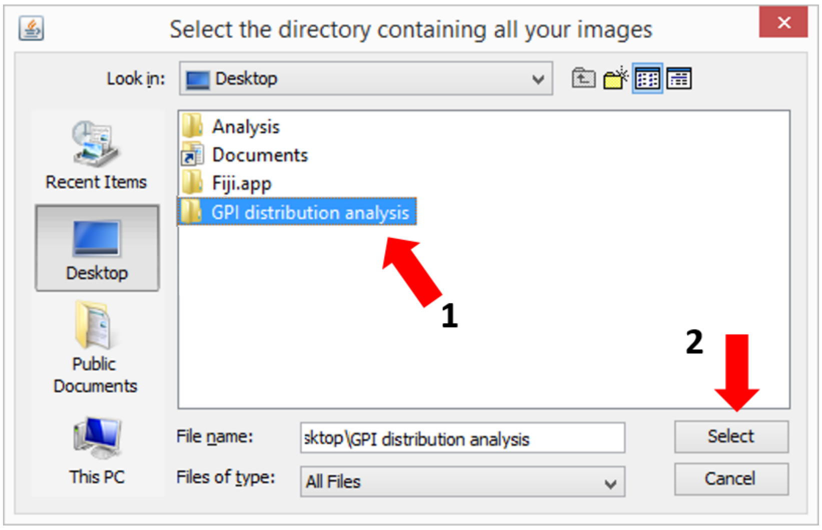

II. A 'Parameters' dialog window will appear. Here, parameters for running the analysis can be set as follows:

1. In the window 'File extension', enter 'txt'.
2. Open the drop-down list 'Image Type' and select 'PALM'.
3. The threshold is fixed to 'Li' by default.
4. In the window 'Pixel Size', enter the number of nanometers per pixel (Pixel Size=106.667, for the provided test data).
5. Click 'OK' to start the analysis.

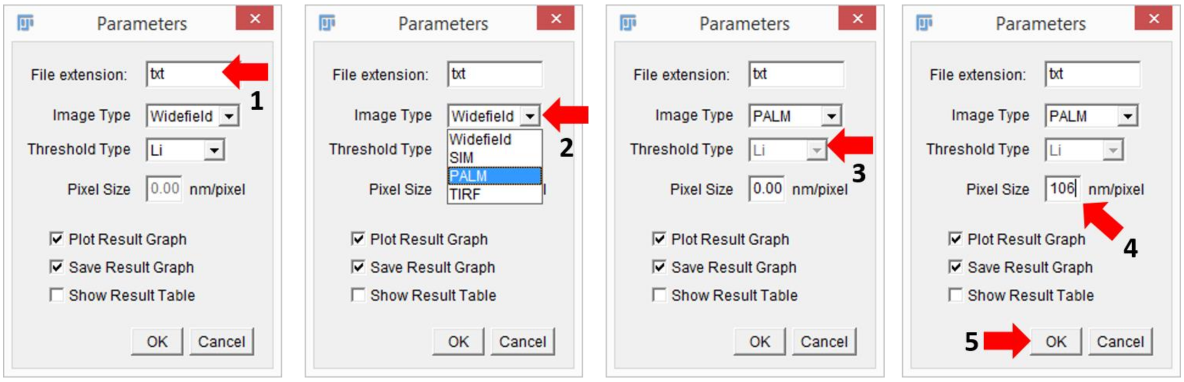

VII. The results include (i) the distance of the shape and scale parameters from the reference random distribution, 'Distance Mean to Random' (this is the measure of distribution inhomogeneity), and (ii) the closest reference distribution for the result, 'Closest Distribution'. The shape and scale parameter distance to the random distribution is 0.49, and 'Cluster 100nm' (diameter) becomes the closest reference distribution.

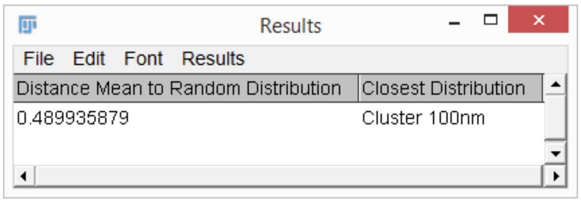

VIII. The QuASIMoDOH Analysis Result plot shows the shape and scale parameters of the reference distributions together with the analysis result. The displayed reference data were obtained from analyzing simulated images with the same density of areas obtained after tile size correction (density equals the number of tiles divided by the area of the image in μm^2). The average density of the analyzed images is provided in the plot title. The reference data in the plot belong to the same density range.

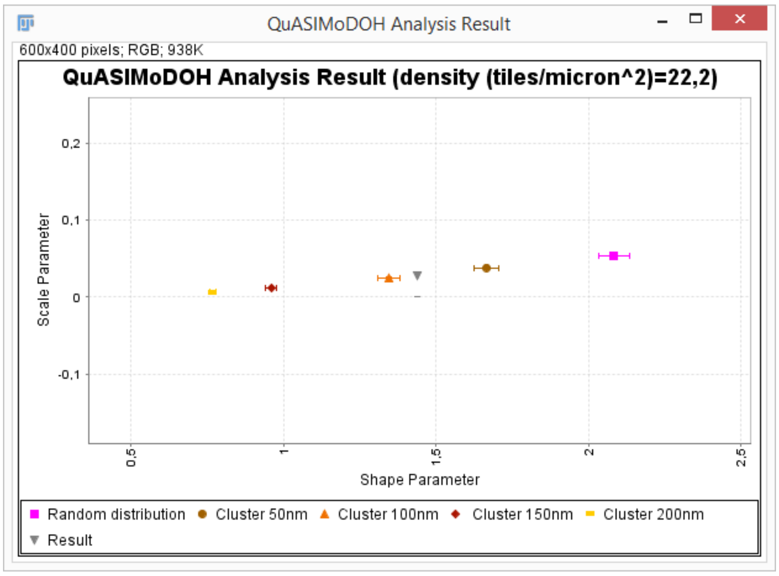

IX. By checking the box 'Show Results Table',

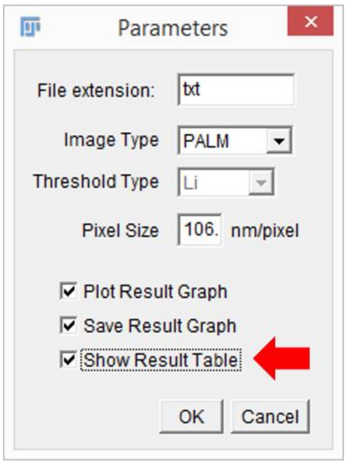

a ‘Log’ file appears:

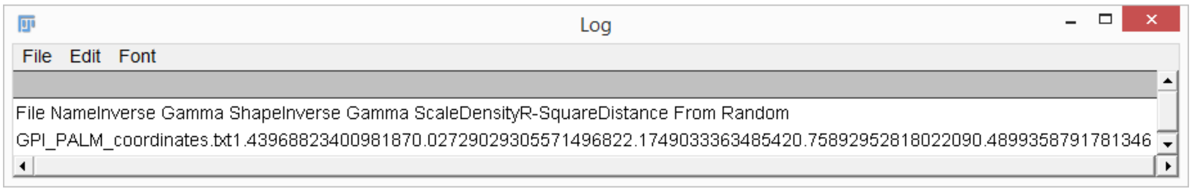

This file contains the following information:

- ‘File Name’: image title.
- ‘Inverse Gamma Shape’: Inverse Gamma shape parameter.
- ‘Inverse Gamma Scale’: Inverse Gamma scale parameter.
- ‘Density’: density of the corrected tile areas.
- ‘R-Square’: coefficient of determination obtained from the fitting of the tile areas histogram using the shape and scale parameters.

C. Local distribution analysis

To run the local analysis, the image to analyze must first be open in Fiji and then ‘Local Analysis’ can be selected from the ‘QuASIMoDOH’ drop-down menu.

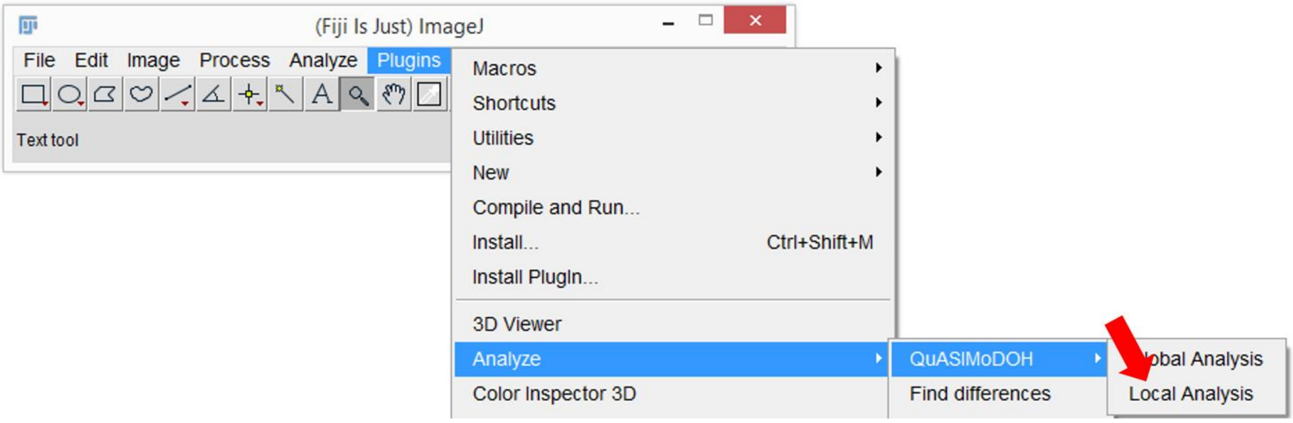

I. A dialog window titled ‘Parameters’ will appear. Set the parameters as follows:

1. Open the drop-down list ‘Image Type’ and select the type of image to analyze based on the microscopy technique used for acquisition.
2. Open the drop-down list ‘Threshold Type’ and select the threshold type previously selected (see above ‘Pre-processing steps for analyzing images with QuASIMoDOH’, step III).
3. Set the ‘Local Analysis Parameters’ by entering the minimum and maximum diameter to use in the dialog boxes ‘Local min diameter for local analysis’ and ‘Local max diameter for local analysis’, respectively (default is 5 μm).
4. Select ‘Draw Legend’ to show the legend of colors.
5. Select ‘OK’ to start the analysis.

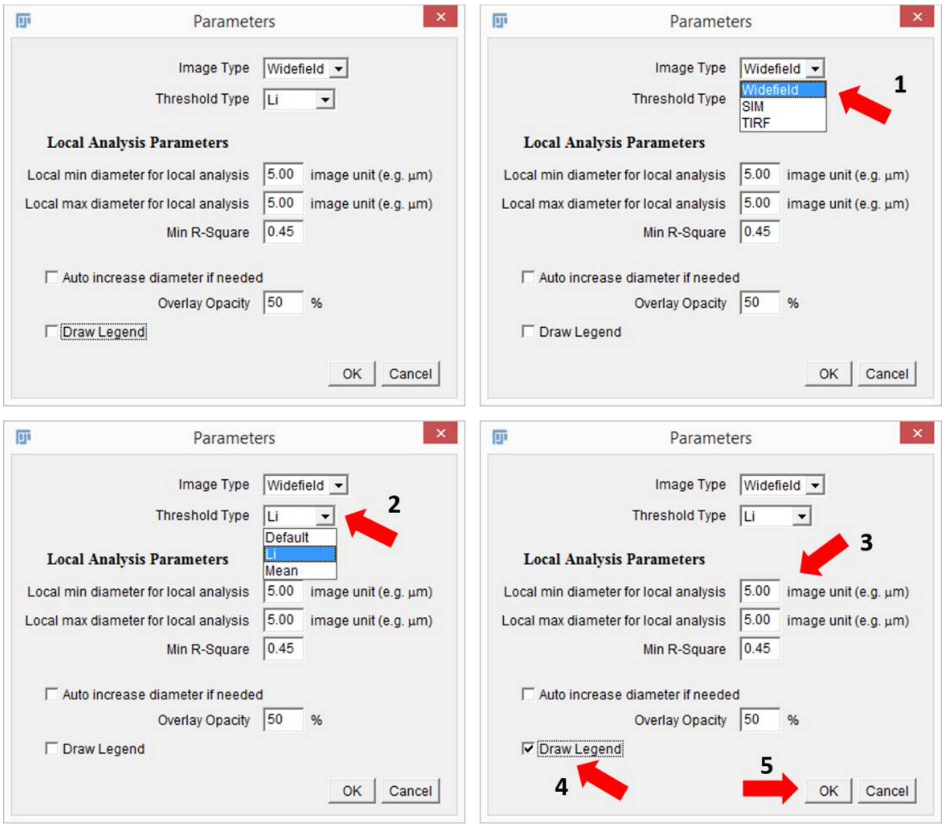

II. The analysis produces a figure titled ‘Local Analysis Map’ where the tiles are colored based on the detected surrounding distribution.

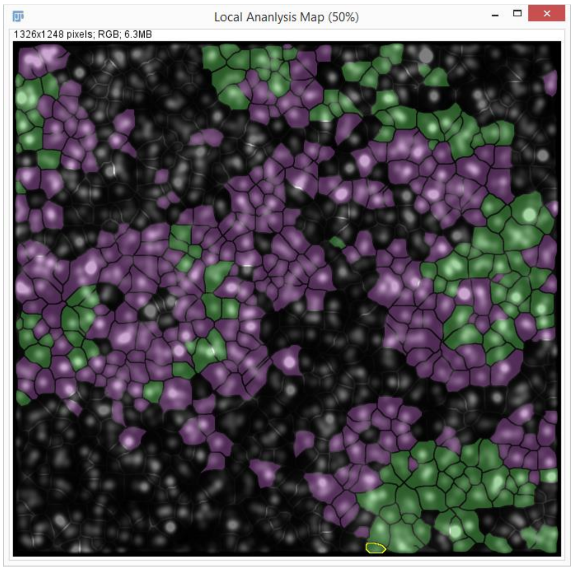

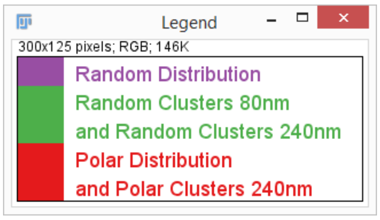

The indices belonging to each tile can be displayed in the image ‘Local Analysis Map’ by checking the box ‘Show all’ on the ROI Manager.

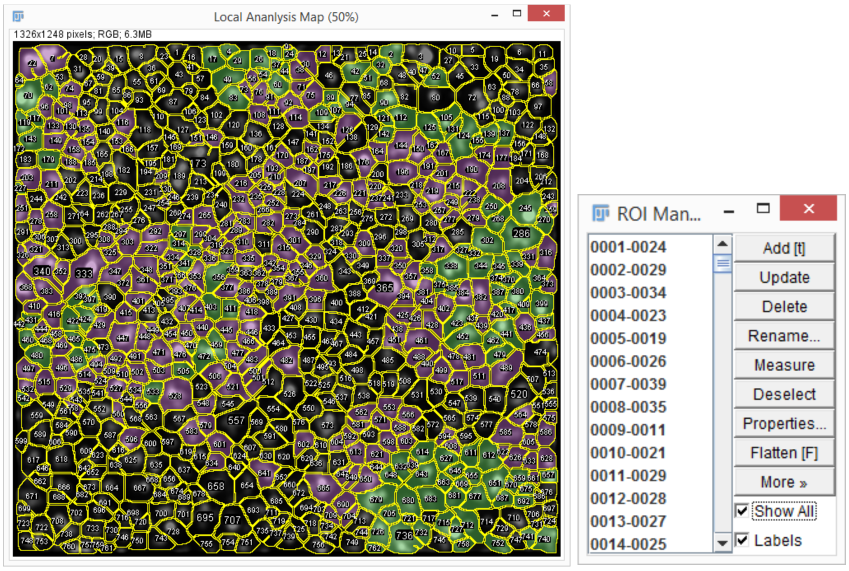

A 'Results' table is also generated for each circle localized tile, and this contains:

- 'ROI Number': index corresponding to the circle localized tile.
- 'Distance to Closest Distribution' of the results of the local analysis.
- 'Closest Distribution': detected closest distribution.
- 'R-Square': coefficient of determination.
- 'Density': density of tiles in the local area.
- 'Diameter': diameter of the circle.
- 'Nr of Tile Areas': number of tile areas obtained after intensity correction.
- 'Inverse Gamma Shape', 'Inverse Gamma Scale' obtained for each local analysis.
- 'ROI indexes': indices of the analyzed tiles in the local area.

Results

File Edit Font Results

ROI Number	Distance to closest Distribution	Closest Distribution	R-Square	Density	Diameter	Nr of Tile Areas	Inverse Gamma Shape	Inverse Gamma Scale	ROI indexes
1	4.765286279	Random Distribution	0.937935100	5.499785090	5.661375661375661 µm	66	12.391715603	2.070616962	8;1;4;16;17;20;26
2	0.736312432	Random Clusters 240nm	0.496587742	4.677878513	5.0 µm	65	5.121762481	0.907422130	2;3;5;10;13;14;12
3	0.128156108	Random Clusters 80nm	0.320971616	4.438817354	5.661375661375661 µm	58	5.649284587	1.069639904	2;3;5;10;14;19;21

If R-Square<0.45 or the distance to the closest distribution is larger than the cutoff (~1), no color is assigned to the tiles (although on the 'Results' table the closest distribution is indicated).

III. To increase the diameter of the local area to analyze:

1. Enter the 'Local min diameter for local analysis' and increase the 'Local max diameter for local analysis' value
2. Select 'Auto increase diameter if needed'
3. Select 'OK' to run the analysis

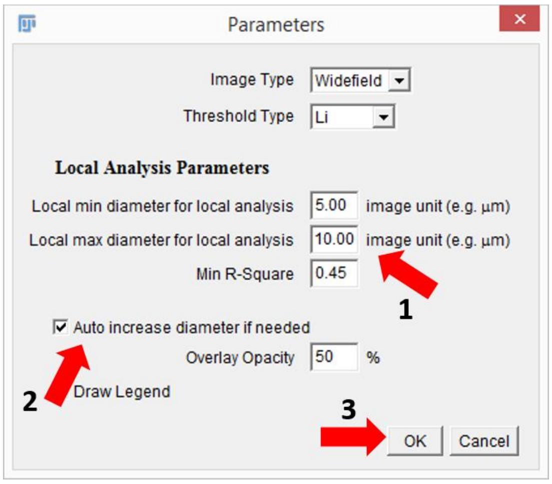

IV. The diameter of the local circle is now increased to reach the maximum diameter.

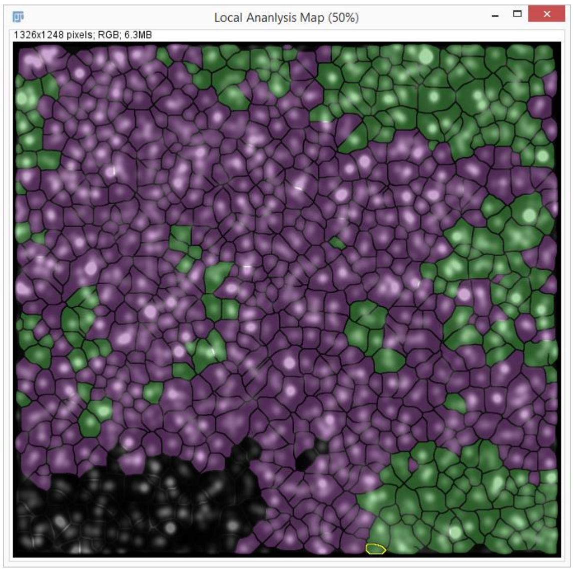

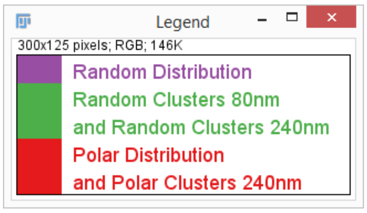

Appendix

Changes in Fiji default settings, possibly due to other plugins, could interfere with QuASIMoDOH analysis. In the case of difficulties, we are happy to provide support.
